# The Transmission Patterns of the Endosymbiont *Wolbachia* within the Hawaiian Drosophilidae Adaptive Radiation

**DOI:** 10.3390/genes14081545

**Published:** 2023-07-27

**Authors:** Renée L. Corpuz, M. Renee Bellinger, Anne Veillet, Karl N. Magnacca, Donald K. Price

**Affiliations:** 1 Department of Biology, Tropical Conservation Biology and Environmental Science, University of Hawaii at Hilo, 200 West Kāwili Street, Hilo, HI 96720, USAdonald.price@unlv.edu (D.K.P.); 2 U.S. Geological Survey, Pacific Island Ecosystems Research Center, P.O. Box 44, Hawaii National Park, HI 96718, USA; 3 Department of Land and Natural Resources, Division of Forestry & Wildlife, Native Ecosystem Protection and Management, Hawaii Invertebrate Program, 1151 Punchbowl Street Rm. 325, Honolulu, HI 96813, USA; knm956@gmail.com; 4 School of Life Sciences, University of Nevada, Las Vegas, NV 89557, USA

**Keywords:** co-phylogenetic reconciliation, co-speciation, evolution, stochastic character trait mapping, horizontal transfer, vertical transfer

## Abstract

The evolution of endosymbionts and their hosts can lead to highly dynamic interactions with varying fitness effects for both the endosymbiont and host species. *Wolbachia*, a ubiquitous endosymbiont of arthropods and nematodes, can have both beneficial and detrimental effects on host fitness. We documented the occurrence and patterns of transmission of *Wolbachia* within the Hawaiian Drosophilidae and examined the potential contributions of *Wolbachia* to the rapid diversification of their hosts. Screens for *Wolbachia* infections across a minimum of 140 species of Hawaiian *Drosophila* and *Scaptomyza* revealed species-level infections of 20.0%, and across all 399 samples, a general infection rate of 10.3%. Among the 44 *Wolbachia* strains we identified using a modified *Wolbachia* multi-locus strain typing scheme, 30 (68.18%) belonged to supergroup B, five (11.36%) belonged to supergroup A, and nine (20.45%) had alleles with conflicting supergroup assignments. Co-phylogenetic reconciliation analysis indicated that *Wolbachia* strain diversity within their endemic Hawaiian Drosophilidae hosts can be explained by vertical (e.g., co-speciation) and horizontal (e.g., host switch) modes of transmission. Results from stochastic character trait mapping suggest that horizontal transmission is associated with the preferred oviposition substrate of the host, but not the host’s plant family or island of occurrence. For Hawaiian Drosophilid species of conservation concern, with 13 species listed as endangered and 1 listed as threatened, knowledge of *Wolbachia* strain types, infection status, and potential for superinfection could assist with conservation breeding programs designed to bolster population sizes, especially when wild populations are supplemented with laboratory-reared, translocated individuals. Future research aimed at improving the understanding of the mechanisms of *Wolbachia* transmission in nature, their impact on the host, and their role in host species formation may shed light on the influence of *Wolbachia* as an evolutionary driver, especially in Hawaiian ecosystems.

## 1. Introduction

The Hawaiian Drosophilidae, long recognized as a striking example of adaptive radiation, are of considerable interest as model systems for understanding the underlying mechanisms of insular speciation [1]. Comprised of up to 1000 species in two major genera (*Scaptomyza* and *Drosophila*), which are believed to have diverged within the Hawaiian archipelago approximately 23.4 million years ago, this taxonomic grouping represents approximately 10% of the insect fauna endemic to the Hawaiian Islands [2,3] and one third of the world’s *Drosophila* species [4]. Numerous mechanisms have been proposed to explain the explosive lineage diversification of Hawaiian Drosophilidae, including isolation, niche availability [5], sexual selection [6], and host plant and substrate shifts [1,3]; however, data are lacking on the potential role of symbiont pressures, despite recognition that symbionts, especially those associated with reproduction, could be a major contributor to insect species formation [7]. In particular, a growing body of empirical evidence suggests that the reproductive endosymbiont *Wolbachia* may play a role in the speciation process of some arthropods [8,9,10], including *Drosophila* [11].

*Wolbachia* is a widespread and common α-proteobacterium (order Rickettsiales) that infects arthropods and nematodes [12]. The relationship between *Wolbachia* and its host can span from parasitism to facultative or obligate mutualism to ultimate mutualism, and in some cases, beneficial and detrimental effects can be simultaneously conferred [13]. *Wolbachia* strains possess a remarkable ability to significantly alter the reproductive functions of its host in ways that serve to enhance the rate of *Wolbachia*’s transmission, be it through cytoplasmic incompatibility, male-killing, feminization of genetic males, increased fecundity of host, and parthenogenesis [13,14]. Thus, through multiple mechanisms, *Wolbachia* possess the means to give rise to reproductive isolation barriers, which could contribute to the divergence of populations into new species [15]. Consistent with that notion, cytoplasmic incompatibility is known to have a direct effect on gene flow and can serve as a mechanism of reproductive isolation between populations [11,16,17]. 

The primary mode of *Wolbachia* infection is vertical transmission to the host’s progeny through the cytoplasm of the egg [14]. Horizontal transmission is believed to occur as well, especially in arthropods, as evidenced by the widespread distribution of *Wolbachia* and its potential to infect new host species [8,18], phylogenetic incongruence between hosts and endosymbionts [12,19]), and evidence for species sweeps [20,21]. In contrast, within filarial nematodes hosts, strict vertical inheritance of *Wolbachia* endosymbionts is evidenced by high levels of co-phylogenetic concordance for certain clades [22,23]. At present, the community-level interactions required for *Wolbachia* strains to be successfully transmitted horizontally and become stable within a new host species remain largely unknown, but in some cases, they are believed to involve transfer through plant tissues or parasitoids of insects [24,25]. 

Molecular methods have been invaluable for the study of *Wolbachia* because of an inability to culture it outside of its host or host cells, owing to its obligate intracellular status [14]. Based on molecular diversity analysis, the genus *Wolbachia* is subdivided into at least 17 possible supergroups [26,27], with terrestrial arthropods most commonly infected by *Wolbachia* belonging to supergroups A and B [28]. Estimates for the incidence of *Wolbachia* in terrestrial arthropod species worldwide range between 40–76% [13,29,30], whereas within-species estimates for *Wolbachia* incidence indicate that infection rates tend to be either exceedingly high (>90%) or considerably low (<10%), depending on the surveyed insect system [13,30]. In native Hawaiian insects, the overall incidence of *Wolbachia* infection at the species level was estimated to be ~14%, and for native Dipteran species (e.g., Drosophilidae and Calliphoridae), 12% [2]. 

Although many mechanisms have been proposed to explain the rapid and extensive diversification of the Hawaiian Drosophilidae, the potential contribution of *Wolbachia* as a driver of speciation and patterns of *Wolbachia* transmission have yet to be examined. Using a single gene marker, *Wolbachia surface protein* (*wsp*), Bennett et al. [2] found the incidence of infection within Hawaiian Drosophilidae, including genera *Drosophila* and *Scaptomyza*, was ~18%. *Wolbachia*’s presence in the Hawaiian Islands, and the knowledge of the potential impacts that it can have on host reproductive strategies, give rise to the question: could *Wolbachia* have played a role in the diversification of the native Hawaiian insects? To begin to address this larger question, in this study we conducted genetic analyses of *Wolbachia* and its Hawaiian Drosophilidae hosts to examine: (1) the *Wolbachia* strain diversity and phylogenetic affiliations; (2) the co-phylogenetic diversification patterns of *Wolbachia* and hosts; and (3) *Wolbachia* host-switching mechanisms through stochastic character trait mapping to construct host ancestral traits.

## 2. Methods 

### 2.1. Biological Specimens Screened for Wolbachia Endosymbionts

The Hawaiian *Drosophila*, many of which are primarily single island endemics that have high levels of host plant specificity, can be subdivided into 4 main groups: *modified mouthparts*, *haleakalae*, *picture wing,* and the AMC clade (comprising the groups *antopocerus*, *modified tarsus,* and *ciliated tarsus*) [31]. The genus *Scaptomyza* is divided into 21 subgenera, 10 of which contain native Hawaiian species [31]. A total of 399 Hawaiian Drosophilidae specimens representing a minimum of 136 species of *Drosophila* and 14 species of *Scaptomyza* collected from Kaua’i (*n* = 50), Lāna’i (*n* = 1), Maui (*n* = 68), Moloka’i (*n* = 17), O’ahu (*n* = 29), and the Island of Hawai’i (*n* = 234) were screened for *Wolbachia* infections (Supplementary S1). A number of undescribed morphospecies in the *Scaptomyza, modified tarsus* and *modified mouthparts* groups of *Drosophila* are included. These Drosophilidae specimens were components of biological collections described in Magnacca and Price [3]. Additional screens for *Wolbachia* infections were conducted from DNA extracts of three species of insects that have invaded the Hawaiian archipelago: *D. suzukii* (*n* = 68 specimens from Kaua’i, O’ahu, and the Island of Hawai’i [32], Supplementary S1), *Aedes albopictus* (*n* = 1, collected on the Island of Hawai’i)*,* and *Culex quinquefasciatus* (collected on the Island of Hawai‘i, sample 6771, [33]. The *Wolbachia* DNA was sourced from whole-body soaks or digests of individual body parts (e.g., genitalia or abdomen) and DNA extractions were performed using Qiagen DNeasy Blood and Tissue Extraction Kits. For notation purposes, *Wolbachia* strains having published lineage assignments are denoted by their host following established practices, e.g., a *Wolbachia* endosymbiont of *Drosophila recens* is written as *w*Rec, or in the case of this study, sample number followed by host species name.

Using seven *Wolbachia* amplification targets (see below) and Sanger sequencing, individual specimens were classified as testing positive for a *Wolbachia* infection if any single amplification target was visible by gel electrophoresis and the sequenced amplicon matched to a *Wolbachia* sequence contained in the National Center of Biotechnology Information (NCBI) GenBank nucleotide sequence repository (approximate search dates: February 2018 to March 2019). 

### 2.2. Wolbachia Gene Sequencing

#### 2.2.1. Amplicon Sequencing and Primer Redesign

We aimed to characterize *Wolbachia* allele diversity and determine the phylogeny of *Wolbachia* by sequencing seven gene targets, five of which are components of the widely-accepted universal Multi-Locus Sequence Typing (MLST) system that assigns *Wolbachia* to a strain type using five housekeeping genes: *coxA* [cytochrome C oxidase subunit A], *fbpA* [fructose-bisphosphate aldolase], *hcpA* [hypothetical conserved protein], *ftsZ* [cell division protein], and *gatB* [aspartyl/glutamyl-tRNA aminotransferase subunit B] [34]. The sixth and seventh gene target, *wsp* [*Wolbachia* surface protein] [34], is duplicated in *Wolbachia* endosymbionts of *Drosophila,* with the paralogous gene denoted *wspB* [*Wolbachia* surface protein (duplicate)] [35]. The gene targets were amplified from DNA extracts using polymerase chain reaction (PCR), visualized using electrophoresis with 1.5% agarose gels, and amplification products purified in preparation for Sanger sequencing on an Applied Biosystems 3500 Genetic Analyzer (see Supplementary Information for details). The chromatograms were viewed and edited using Sequencher version 5.2.4 (Gene Codes Corporation). Based on chromatogram visualization, samples that showed evidence of a double *Wolbachia* infection were sequenced from clones generated with a TOPO-TA Cloning Kit using One Shot Chemically Competent TOP 10 *Escherichia coli* cells.

Preliminary amplification results showed high rates of amplification failures; therefore, to increase primer specificity, we redesigned primers for supergroups A and B in insect hosts. Primer re-design efforts utilized a combination of sequence data obtained from: (a) the *n* = 31 sequences generated in this study using original primer pairs, (b) *w*Drosophila gene sequences (*n* = 195) downloaded from the National Center for Biotechnology Information (NCBI), and (c) nucleotide sequences in silico extracted from five *w*Drosophila reference genomes belonging to supergroups A and B (Table 1). Those included: *Wolbachia* endosymbionts of *D. recens* (*w*Rec), *D. melanogaster (w*Mel), *D. simulans* (*w*No), *D. suzukii* (*w*Suzi), and *D. ananassae* (*w*Ana). Target regions within genomes were identified by BLASTn (v 2.2.30) using the 231 available sequences as queries, with the per-gene number of query sequences ranging from three (*fbpA*) to 141 (*wsp*) (accessions available from Supplementary S2). The BLASTn hits were filtered using a threshold *e*-value <0.001, and gene target regions were excised in silico along with 200 base pair regions flanking the 5′ and 3′ reading frames. Next, multiple sequence alignment was conducted for each gene in MEGA7 [36] and candidate primers were designed across sites internal or external to the MLST gene targets. Finally, all pairwise combinations of redesigned and original primers were tested for improved amplification and sequencing efficiency (see Supplementary Information). These efforts increased data for *hcpA*, *fbpA*, and *ftsZ* by 54 sequences obtained from 93 additional amplifications, yet the re-designed primers failed to improve amplifications for genes *coxA* and *gatB*. The overall poor amplification success for *wsp* and *wspB* (consistent with findings by Wu et al. [35]), led to the exclusion of those two genes for phylogenetic and strain typing analyses, while poor amplification of *gatB* led to the exclusion of that gene from phylogenetic analysis. The primer design strategy, PCR conditions for the original and modified primers, primer sequences, and those re-designed for this study are available from Supplementary Information.

#### 2.2.2. *Wolbachia* Sequence Datasets

The final *Wolbachia* dataset included MLST genes amplified and sequenced from DNA extracts of native *Drosophila* spp., *Scaptomyza* spp. and invasive species *D. suzukii*, *C. quinquefasciatus*, and *A. albopictus* hosts as described above, plus published *Wolbachia* nucleotide sequences downloaded from the MLST database or extracted from genomes (Table 1). The published sequences were used as references for assigning *Wolbachia* alleles to supergroups and used as outgroups in phylogenetic reconstructions, plus represent *Wolbachia* endosymbionts of *Drosophila* hosts and mosquitoes sampled from around the world. After aligning sequences in MEGA7 using the ClustalW algorithm [36], the sequences were manually adjusted to ensure that all codons were in the correct reading frame and trimmed so that each sequence began and ended with a codon. The *Wolbachia* sequence data generated for this study are available from Supplementary Datafile S1.

#### 2.2.3. *Wolbachia* Supergroup Designation

Previous studies have shown that phylogenetic clustering of individual MLST genes is sufficient for the classification of *Wolbachia* alleles into supergroups A and B [45]. To evaluate if sequence data from re-designed MSLT primers performed similarly well, we reconstructed single-gene phylogenies using our sequence data and eight published reference sequences. These included the following: supergroup A, *w*Mel, *w*Suzi (strain valsugana), endosymbionts of *D. simulans* (*w*Ha) and *A. albopictus* (*w*AlbA); supergroup B, *w*No, endosymbionts of *C. quinquefasciatus w*Pip (sample 6771, [33]); and supergroup D and F outgroup sequences from *Wolbachia* endosymbionts of *B. malayi* (nematode, *w*Bm) and *C. lectularius* (bed bug, *w*Cle) (Table 1). Phylogenetic patterns for individual gene trees were inferred using a Bayesian methodology implemented in MrBayes (v3.2.5) [46] and the Maximum-Likelihood methodology implemented in RAxML (v1.5b2) [47].

#### 2.2.4. *Wolbachia* Strain Typing

The MLST strain typing protocol established by Baldo et al. [34] defines an ‘allele’ as a nucleotide sequence that differs by at least 1 nucleotide base, and it classifies a ‘strain’ as unique if any individual possesses at least one different allele across any of the five loci, with data at all five loci required for strain assignment. We were unable to apply established MLST conventions (http://pubmlst.org/wolbachia; accessed on 1 July 2017; [34]) for allele and strain categorizations for two reasons: the universal MLST primer sets failed to produce amplifications at all five loci across the majority of our samples, and the amplicon products produced with redesigned primers did not span the full-length of MLST gene sequences. Therefore, we categorized each allele by supergroup affiliation based on single-gene trees and assigned each allele an arbitrary numeric code, which permitted comparison of allele variability and supergroup designations across species. 

#### 2.2.5. Phylogenetic Reconstructions

Evolutionary relationships and genetic similarity of *Wolbachia* strains can be inferred through phylogenetic analyses, and phylogenetic concordance between host and symbiont phylogenies can indicate co-speciation or horizontal transfer events between the two groups. We performed phylogenetic reconstruction for *Wolbachia* strains and their hosts, including Hawaiian Drosophilidae, invasive *Drosophila* flies and mosquitoes, and outgroup taxa, using Bayesian methodologies implemented in MrBayes (v3.2.5) [46] and the Maximum-Likelihood methodology implemented in RAxML (v1.5b2) [47]. Model selection and procedures are available from Supplementary Information, and the final set of trees were visualized and edited in FigTree v1.4.3 [48].

#### 2.2.6. *Wolbachia* Phylogenetic Signals

The five *Wolbachia* MLST gene targets were not successfully amplified in all samples. Therefore, to assess the impact of missing sequences on phylogenetic reconstructions, we examined concordance of *Wolbachia* supergroup designation based on single and concatenated gene trees. Phylogenetic reconstructions for 5-, 4-, and 3-gene MLST data sets revealed that strain assignments and tree topologies were consistent in nearly all cases (see Supplementary Information); therefore, we applied the 3-gene MLST dataset for co-phylogenetic reconciliation analysis and stochastic character trait mapping. 

#### 2.2.7. Host Sequence Data Set 

Phylogenetic reconstruction for Hawaiian *Drosophila* and *Scaptomyza* was inferred using a sequence data set previously shown to produce a well-resolved Hawaiian Drosophilidae phylogeny [3]. However, we used only four of the five genes published in that study (*EF1g* [elongation factor 1-γ], *Gpdh* [glycerol-3-phosphate dehydrogenase], *Pgi* [phosphoglucose isomerase], *Yp2* [yolk protein 2]). The gene *Fz4* (*frizzled* 4) was excluded because of high levels of missing data in the original published dataset, which had negligible effects on the tree topology (compared to [3]). Only Hawaiian Drosophilids having confirmed *Wolbachia* infections with three or more sequences were utilized for phylogenetic reconstructions, along with host sequences obtained by a BLASTn search of genome contents for *D. suzukii*, *D. melanogaster*, *D. simulans*, and two mosquito species, *A. albopictus* and *C. quinquefasciatus* (accessions available from Table S1). Searches for genes in mosquitoes recovered genes *EF1g*, *Gpdh*, and *Pgi* but not *Yp2* (or *Fz4*). The concatenated host sequence data set totaled to 1812 bp across the 4 genes (*EF1g* [507 bp], *Gpdh* [363 bp], *Pgi* [306 bp], *Yp2* [636 bp]). 

#### 2.2.8. Co-phylogenetic Assessment of Host Species and *Wolbachia* Strains 

To evaluate biological events that might influence associations between host and symbiont phylogenies, we conducted co-phylogenetic reconstruction analyses for *Wolbachia* and the Hawaiian Drosophilidae, as well as *Wolbachia* and the 2 mosquito host species collected on the Island of Hawai‘i. By considering five possible biological events (co-speciation, duplication, duplication and host switch, loss, and failure to diverge) and applying each a cost, JANE [49] used a heuristic approach to evaluate and find minimal cost solutions that best explain associations between host and endosymbiont phylogenies [49]. Two models were considered by setting the co-speciation cost parameter to 0 or 1, while keeping all other parameters fixed as follows: loss, failure to diverge, and duplication were each set to a cost of 1, and the parameter duplication and host switch was set to a cost of 2 [49,50]. The genetic algorithm parameters were set to a population size of 23 and the number of generations set to 45, as suggested by Conow et al. [49]. Additional statistical parameters included selecting the random tip mapping procedure with 1000 replicates. Data inputs included host and endosymbiont trees based on Bayesian inference using the codon position data set for the host species and the 3-gene, gene + codon position data set (*coxA*, *hcpA*, and *ftsZ*) for *Wolbachia* (see Supplementary Information for justification). Additionally, a co-phylogenetic tanglegram was produced using the cophylo function in the phytools v0.6-44 package in R [51]. 

#### 2.2.9. Stochastic Character Mapping

Potential host-switching mechanisms were evaluated using stochastic character trait mapping [52], which characterizes associations between *Wolbachia* phylogenies and host species characteristics. When co-speciation can be explained by a particular host trait, evolutionarily conserved characters of the hosts are reflected in the phylogenetic reconstruction of their endosymbionts. Data inputs included three host species traits, island of collection, host plant families, and preferred ovipositional substrate [3], with analyses conducted using the *Wolbachia* 3-gene and gene + codon position data set (*coxA*, *hcpA*, and *ftsZ*) (see Supplementary Information for justification). The contemporary host character traits are depicted on branch tips as a pie chart, with a priori known character traits indicated by 1.0 probability (i.e., 100%) and unknown character traits depicted as equal probability across all possible categories (e.g., 0 ≤ *x* ≤ 1), with the sum of all character state probabilities equaling 1. The internal nodes (also a pie chart) depict the posterior probability of each host character trait being the ancestral state, which reflects the strength of the association between that host trait and the endosymbiont phylogeny. This analysis was performed using phytools v0.6–44 package in R [51]. A total of 225 stochastic character maps were constructed using a model of even rates, as it was indicated to be the best model based on the computed Akaike Information Criterion (AIC) values using the phytools fitMK function (Table S3).

## 3. Results

### 3.1. Incidence of Wolbachia Infection 

Among the 150 species of Hawaiian Drosophilidae screened (including undescribed morphospecies), *Wolbachia* infections were confirmed for 30 species (20.0% species infection rate), and across the entire data set, infections were confirmed for 41 of 399 specimens (10.3% overall specimen infection rate) (Table S2). At a genus level, infection frequencies were higher in *Scaptomyza* (seven of 14 species screened, 50.0%) than *Drosophila* (23 of 136 species screened, 16.9%). An additional 24 Hawaiian Drosophilidae specimens belonging to 17 species (including five undescribed) showed evidence of infection by presence of PCR bands, but infection by *Wolbachia* could not be confirmed owing to the amplicons failing Sanger sequencing. Had those samples been included in the *Wolbachia* infection tally (65/399), the overall infection rate would increase to 16.3%. Some insights into the variability of infection status by species (and sequencing success) can be gleaned from species having data from multiple samples. For example, among 13 species with five or more samples screened (excluding the taxa resembling *D. basimacula*, a complex of undescribed species), the proportion of within-species infections ranged from 0% to 29% (Table 2). A caveat to these findings is that within-species infection rates are known to vary widely (i.e., 10–90%), and a sample size larger than what was available in our specimen collection is required for a robust assessment of infection rates. Screens of the invasive *D. suzukii* indicated that 8 of 68 (11.8%) individuals possessed a *Wolbachia* infection, and that 20 additional individuals may have been infected based on PCR amplification alone. A record of PCR amplicons and sequencing is provided in Table S2.

### 3.2. Wolbachia Strain Typing and Supergroup Designations

A complete MLST profile (5 genes: *coxA*, *fbpA*, *gatB, hcpA*, and *ftsZ*) was obtained for *Wolbachia* endosymbionts of only 9 individual Hawaiian Drosophilidae, all of which belonged to supergroup B, plus *w*Bm and *w*Cle outgroup taxa belonging to supergroups D and F. The *gatB* gene failed PCR amplification across the majority of individual Drosophilidae and was not recovered from endosymbiont genomes belonging to hosts *D. suzukii*, *A. albopictus*, and *C. quinquefasciatus*, leaving only genes *coxA*, *fbpA*, *hcpA*, and *ftsZ* available for analytical inferences across the majority of *Wolbachia* datasets. Individual-gene phylogenetic reconstructions of *Wolbachia* based on *coxA*, *fbpA*, *hcpA*, and *ftsZ* gene sequences (*n* = 46, 33, 44, and 28 sequences, respectively) showed strong support for the clustering of alleles by supergroup, although supergroup sister status and placement relative to the supergroups D and F outgroups was inconsistent across trees, and placement of some individuals within supergroup clusters varied slightly (Figures S1 and S2, Bayesian and Maximum-Likelihood trees).

Phylogenetic reconstructions of *Wolbachia* based on the concatenated data set comprised of *coxA*, *hcpA*, and *ftsZ* genes, and the 25 individuals with data available at all three genes (including outgroups), showed clear separation between supergroups A and B (Figure S3), consistent with the four-gene dataset (Figure S4). However, the three-gene dataset showed supergroup B placed interior to supergroup A (instead of sister), possibly driven by inclusion of the additional set of *Wolbachia* sequences (247*w*D. engyochracea, 266*w*D. Hawaiiensis, and the invasive *w*Alb collected on the Island of Hawai‘i) that had conflicting supergroup assignments and were positioned intermediately between supergroups A and B. Given that the three-gene data set recovered a reasonable degree of phylogenetic structure, and allowed use of the maximum available data, we selected that dataset, using the Bayesian method and partition scheme ‘gene and codon position’, for co-phylogenetic reconciliation analyses and stochastic character trait mapping. The analysis method (Bayesian versus Maximum-Likelihood analyses (Figures S3 and S4) had little effect on tree topologies, and no significant statistical differences were detected between their top likelihood scores (See Supplementary Information for model selection justification).

### 3.3. Strain Typing

A total of 41 Hawaiian Drosophilidae were confirmed as having *Wolbachia* infections, with four individuals (w16 *D. large spots*, w208 *D. apodasta*, w215 *D. nr. perissopoda #1*, w250 *D. engyochracea*) doubly infected (Table 3, Table S2). Among the 44 *Wolbachia* typed with MLST markers, a minimum of 27 unique strains were present based on *Wolbachia* allelic diversity analysis. This minimum number of strains is conservative because only nine *Wolbachia* (representing seven unique strains) could be sequenced across all five gene targets (Table 3). Patterns of infection varied by species, for example, one individual of *D. engyochracea* was doubly infected, one was single-infected, and one showed a PCR amplification, but the PCR product failed to sequence. The majority (30/44, 68%) of *Wolbachia* alleles belonged to supergroup B across all loci (Table 3), based on individual gene trees, while only five (5/44, 11%) belonged to supergroup A, including two from within the double-infected *D. engyochracea*. A modest amount (9/44, 20%) of *Wolbachia* strains were characterized as having supergroup A and B alleles that conflicted across individual gene trees, including two *Drosophila* spp. (of four) with double-infections. The *hcpA* allele 11 was responsible for seven of the nine observed A/B allelic conflicts, and one allele (allele 3) did not clearly assign to supergroup A or B in the single-gene phylogeny (Figures S1 and S2). Additional patterns of interest were that the *hcpA* allele 14 was shared by the *Wolbachia* endosymbiont of native *S. undulata* and invasive *D. suzukii* hosts, and that allele 13 was detected in *Wolbachia* of two distantly related invasive host flies sampled in Hawai‘i: *D. suzukii* and *D. simulans*. For *C. quinquefasciatus* host specimens collected on the Island of Hawai‘i, South Africa and Sri Lanka, only a single strain of *Wolbachia* was detected. Two alleles, at two genes (*coxA*, allele 13; *hcpA,* allele 11), were detected in *Culex* and also >10 Hawaiian Drosophilidae, but in no cases were those two alleles observed in the identical combination in flies as was observed in mosquitoes. Conversely, *w*Alb, isolated from the *A. albopictus* specimen collected on the Island of Hawai‘i(sequenced for this study), had no alleles in common with the other *w*Alb sample [34] or even with any Hawaiian Drosophilidae. A limitation to our study is that we were unable to match allele names to those contained in the online MLST database curated by Baldo and colleagues (http://pubmlst.org/wolbachia/, [34])) because we had to use redesigned primers to successfully sequence the genes in Hawaiian *Drosophila*. Therefore, the gene sequences in our dataset are of different sequence lengths compared to the MLST database and we could not determine if the alleles that were sequenced in this study are “novel” to Hawai‘i or to what parts of the world they are most similar.

Patterns of *Wolbachia* strain diversity corresponded to host relatedness in some, but not all cases. Two closely related, sympatric host species, *D. hawaiiensis* and *D. engyochracea* were possibly infected with the same, or if not the same, a similar *Wolbachia* strain (at 3-identical alleles, Table 3). Furthermore, within the same population, an additional *D. engyochracea* specimen was doubly infected with one *Wolbachia* strain identical to *D. engyochracea* and *D. hawaiiensis* (at two alleles), plus a second strain with two unique alleles, both belonging to the uncommon supergroup A. Evidence of infection by identical *Wolbachia* strains (at five loci) was found for the distantly related host species *S. caliginosa* and *D. seclusa*, both collected on the island of Hawaii. Interestingly, it was also found that the five members of the *D. basimacula/perissopoda* “bristle tarsus” complex were each infected by a different *Wolbachia* strain, while a sixth (*D.* nr. *perissopoda* #5) was not infected. Each is only represented by one or two individuals, but the strains appear to be the same within each taxon.

### 3.4. Phylogenetic Reconstruction Analysis

Phylograms for Hawaiian Drosophilidae host species showed nearly identical topologies between inferences made with Bayesian and Maximum Likelihood analyses (Figure S5) and were approximately concordant with the Hawaiian Drosophilidae phylogram previously published by Magnacca and Price [3]. The only discrepancy is the placement of the modified mouthparts group (represented here by *D. nigrocirrus* and *D.* “large spots”) as sister to the *picture wing* group with the AMC clade basal, rather than with the *picture wing* group basal as they were found. However, Magnacca and Price [3] noted that the phylogenetic position of the modified mouthparts and AMC clade (outgroups) relative to the *picture wing* species group were not well supported, and in fact the arrangement found here is the same as in their analysis using BEAST. The addition of *A. albopictus* and *C. quinquefasciatus* had minimal effect on tree topology.

### 3.5. Co-Phylogenetic Reconciliation

**Figure 1:**
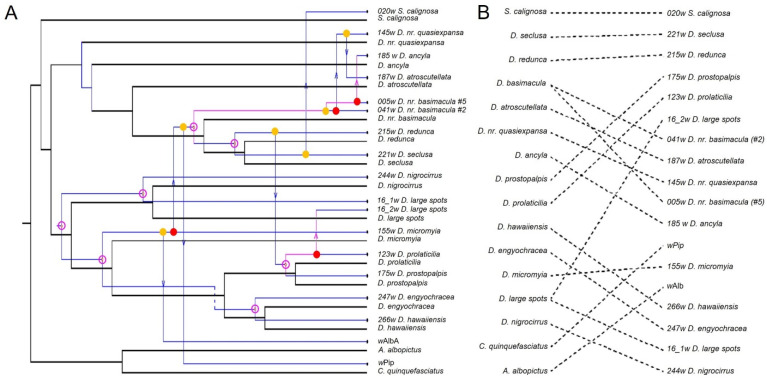
(**A**). Co-phylogenetic reconciliation analysis for Hawaiian Drosophilidae and two species of invasive mosquitoes and their *Wolbachia* endosymbionts based on the following cost scheme: co-speciation: 1; duplication: 1; duplication and host switch: 2; loss: 1; failure to diverge: 1. The estimated biological events that best describe the data are depicted on the phylogeny [open circle: co-speciation; closed circle: duplication; closed circle with arrow: duplication and host switch; dashed line: loss]. Red indicates that the event is optimally placed, whereas yellow indicates that another placement exists that is equally valid. (**B**). A tanglegram depicting the co-phylogenetic relationship between the Hawaiian Drosophilidae and invasive mosquito phylogeny (**left**) and their *Wolbachia* endosymbiont phylogeny (**right**).

The co-phylogenetic reconciliation analysis run in JANE [49] determined that the optimal solutions consisted of two main biological events: co-speciation and duplication with host switches (Table 4, Figure 1A). The co-phylogenetic reconstructions for the dataset consisting of only Hawaiian Drosophilidae and their *Wolbachia* endosymbionts resulted in identical optimal solutions, regardless of co-speciation being assigned a cost of 0 or 1, and the pattern of events was similar, differing only slightly by the projected timing of events (Figure S6, Panels A and B). When invasive mosquitoes collected in Hawaii and their *Wolbachia* endosymbionts were added to the data, the optimal solutions differed slightly by the number of each event, and the optimized cost between the two models differed significantly (*p* < 0.01), indicating that co-speciation has a significant effect on the overall model (Table 4, Figure 1A and Figure S6). Lastly, a tanglegram illustrates that co-phylogenetic relationships between *Wolbachia* and its host show patterns consistent with both co-evolution (parallel connections) and horizontal transfer (crossed lines) (Figure 1B).

### 3.6. Stochastic Character Trait Mapping

**Figure 2:**
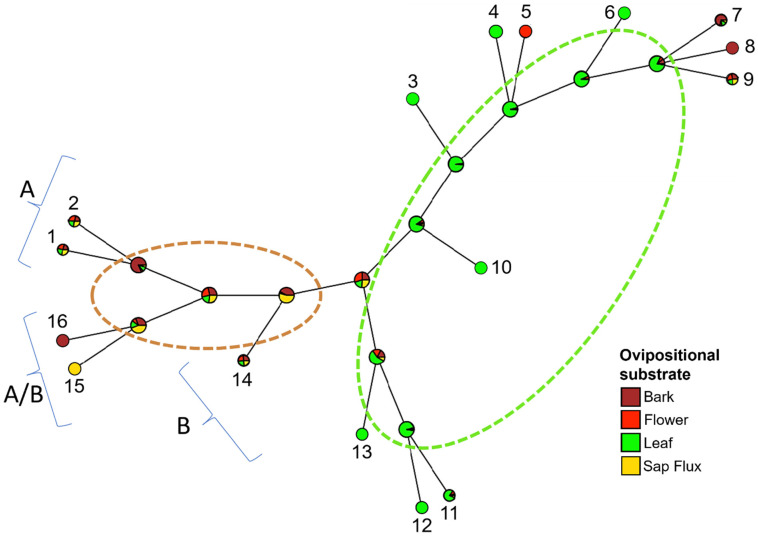
Stochastic character mapping of the Hawaiian Drosophilidae (*Drosophila* and *Scaptomyza*) host species’ ancestral trait ‘ovipositional substrate’ (categories: bark, flower, leaf, and sap flux) mapped to an unrooted *Wolbachia* phylogeny. External nodes depict host trait assignments: solid = known, equal proportions = unknown. Interior nodes represent posterior probabilities that the host’s character trait is ancestral and congruent with the phylogenetic signal of the strain of their *Wolbachia* endosymbiont. Key to *Wolbachia* found in host individual listed in Supplementary S1 (sample number, species name): (1) 244 w *D. nigrocirrus*, (2) 16_1w *D. “*large spots” (double-infected strain A), (3) 185w *D. ancyla*, (4) 221w *D. seclusa*, (5) 20w *S. caliginosa*, (6) 216w *D.* nr. *redunca,* (7) 175w *D. prostopalpis*, (8) 123w *D. prolaticilia,* (9) 16_2w *D. “*large spots” (double-infected strain B), (10) 5w *D.* nr. *basimacula #5*, (11) 187w *D. atroscutellata*, (12) 145 w *D. quasiexpansa*, (13) 41w *D.* nr. *basimacula #2*, (14) 155 w *D. micromyia,* (15) 266w *D. hawaiiensis*, and (16) 247w *D. engyochracea*. Two strains belong to supergroup A, two were intermediate A/B, and all other strains belong to supergroup B.

The modeled ancestral state of host ovipositional substrate showed high posterior probabilities (depicted on the interior node) when mapped to the unrooted *Wolbachia* phylogeny, reflecting a phylogenetic signal for this character among similar *Wolbachia* strains (Figure 2). The bark and sap flux ancestral traits were, for the most part, conserved among supergroups A and A/B, while the bulk of supergroup B *Wolbachia* was affiliated with the trait leaf. In contrast, little support was evident for host trait associations to *Wolbachia* phylogenies for the host traits island of collection and host plant family (Figure S7).

## 4. Discussion

Our assessment of *Wolbachia* within the Hawaiian Drosophilidae family contributes to the understanding of endosymbiont transmission and its potential role in speciation. Using a modified MLST strain typing protocol, and through phylogenetic analyses, we found evidence for both coevolution and horizontal transmission of *Wolbachia* within *Drosophila* sampled across the Hawaiian archipelago. Our study complements the singular previous broad-scale study of *Wolbachia* within natural populations of Hawaiian insect taxa by Bennett et al. [2], in which strain diversity was characterized using a single gene marker, *wsp*. These studies differed by taxonomic scope, in that our primary focus was to investigate *Wolbachia* strain diversity among members of native Hawaiian Drosophilidae (and select invasive insects), and we used a modified version of the MLST strain typing scheme developed by Baldo et al. [34]. Despite study design differences, findings across studies were largely concordant, with Bennett et al. [2] determining the species-level incidence of *Wolbachia* infection for native Hawaiian Drosophilidae to be 18.1%, compared to our finding of 20.0%. Across all samples screened, we found an infection rate of 10.3%, which is lower than Bennett et al.’s [2] incidence of infection at 18.1%. That difference in infection rate can be attributed to the sampling of different taxa, along with uneven sample numbers within individual species. We caution that many species considered in this study were represented by only a single individual; thus, infection status is not representative of the species as a whole. Indeed, we found strong differences in percent infection rate within individual species having data available for five or more individuals. Additionally, although our efforts to re-design *Wolbachia* MLST primers improved amplification efficiency and increased the number of confirmed infections, the amplification and sequencing of *Wolbachia* alleles still proved to be difficult and infection rates may thus be an underestimate. A few of the species (namely *D. claytonae* and *D. setosifrons*) are also represented only by older specimens with poor DNA extractions, which may not have yielded enough to detect *Wolbachia*. If specimens with PCR bands only (absent sequencing results) were to be counted as positive infections, the incidence of *Wolbachia* at both the species and individual level would increase to 28.1% and 16.3%, respectively.

Between supergroups A and B, the majority of *Wolbachia* strains in Hawaiian Drosophilidae were determined to belong to supergroup B (at 68%), consistent with previous screens in native Hawaiian insect taxa, using *wsp*, at ~75% [2]. Among the species included in Bennett et al.’s [2] study, and also screened here, the *Wolbachia* supergroup designations were concordant for endosymbionts of *D. basimacula*, *D.* nr. *basimacula*, *D. redunca*, and *D. ancyla*, which harbored *Wolbachia* from supergroup B, and *D. nigrocirrus*, which harbored *Wolbachia* from supergroup A. With regards to invasive *Drosophila,* Bennett et al. [2] found that *D. suzukii* was infected only by *Wolbachia* belonging to supergroup A, whereas we found individuals harboring infections belonging to supergroups A (*n* = 5) and B (*n* = 3). Interestingly, we observed that a *Wolbachia* infecting a *D. suzukii* individual collected from Hawai‘i shared at least two identical alleles (*coxA* and *hcpA*) with the non-native species *D. simulans* that was also collected from Hawai‘i by Ellegaard et al. [38]).

### 4.1. Mechanisms of Wolbachia Transmission

In the case of purely vertical transmission of *Wolbachia* within the Hawaiian Drosophilidae, the expectation is that *Wolbachia* strains would be most similar between closely related host species and that phylogenetic reconstructions of the host and endosymbiont would be fully congruent [18]. The alternative hypothesis is that host-switching may play a role in transmission, in which case host and endosymbiont phylogenies would be discordant. Using co-phylogenetic reconciliation analysis, we found that optimal solutions generated by JANE consistently showed co-speciation (i.e., vertical transmission) and duplication with host switching (i.e., horizontal transmission) events as significant parameters despite the costs associated with them. Further evidence for both scenarios—vertical and horizontal transmission—are evidenced through strain typing results. For example, the distantly related species *D. seclusa* and *S. caliginosa* possessed seemingly identical *Wolbachia* strains, and conversely, individual hosts belonging to the same species harbored differing *Wolbachia* strains (e.g., *D. engyochracea*). Mechanisms for horizontal transmission are suggested by stochastic character trait mapping results, which revealed a positive association between phylogenetic patterns of *Wolbachia* and their hosts’ ancestral trait preferred host ovipositional substrate, a trait that is more evolutionarily conserved than affiliations with host plant families [3,31]. For preferred ovipositional substrate, in general, Hawaiian Drosophilidae from the genus *Scaptomyza* use flowers or rotting fruits (as well as many unusual substrates, such as living *Cyrtandra* leaves), the AMC clade (i.e., *antopocerus*, *modified-tarsus*, *ciliated-tarsus*) utilizes rotting leaves, the *picture wing* species group uses rotting bark or sap-flux, and the *modified mouthparts* clade (e.g., *D. nigrocirrus* and *D. large spots*) uses a range of ovipositional substrate types [31]. High posterior probabilities for ancestral states of host ovipositional substrate indicated associations between the trait ‘bark’ and ‘sap flux’ for supergroups A and A/B and the trait ‘leaf’ for supergroup B. This pattern was consistent even for the single *D. large spots* specimen doubly infected by *Wolbachia* strains belonging to supergroups A and B. Notably, the only other *Wolbachia* belonging to supergroup A isolated from Hawaiian *Drosophila* was isolated from *D. nigrocirrus*, also a member of the *modified mouthparts* sub-group. The host plant and substrate are unknown for both of these species. Bennett and colleagues [2] noted that phylogenetically, *wsp* alleles amplified from Hawaiian taxa tended to group closely together, and they found evidence for sharing of identical or similar *wsp* alleles between close and distantly related Hawaiian insect species. They postulated that this observation can be explained by *Wolbachia* infections persisting through speciation, as well as horizontal transmission occurring between host taxa.

An association of *Wolbachia* supergroup B with the decaying leaf substrate could play a role in one of the evolutionary puzzles of Hawaiian Drosophilidae, namely, why there are so many closely related, sympatric species utilizing the same host substrate. This is most readily seen in the *spoon tarsus* subgroup on Hawai’i and the *bristle tarsus* subgroup on Kaua’i. The latter is represented here by six members of the *D. basimacula–perissopoda* species complex, which can be distinguished by the number and arrangement of thickened bristles on the modified front tarsus of the male. Each was found to carry a different strain of *Wolbachia*, or none. Novel infection or loss of infection may initiate the localized equivalent of “founder events”, leading to rapid speciation and maintenance of species boundaries when combined with the sexual selection for which Hawaiian *Drosophila* are well known [53].

Consistent with our findings, plants are thought to play key roles in the horizontal transmission of *Wolbachia* strains between infected and uninfected individuals, as well as between diverse insect species. For example, Sintupachee et al. [54]) found that distantly related species of arthropods found to co-occur on pumpkin leaves harbored *Wolbachia* with similar *wsp* sequences, and Li et al. [25] showed under a controlled experimental laboratory setting that a stable *Wolbachia* infection could be attained by uninfected whitefly individuals through feeding on the same leaf substrate previously exposed to *Wolbachia* infected individuals. In that study, *Wolbachia* was documented as dispersing to adjacent leaves within just a few days of the initial plant infection, where it remained within the phloem of the plant for a minimum of 50 days [25]. In Hawaiian insects, Bennett et al. [2] found that nearly identical *Wolbachia wsp* alleles were shared between some Diptera species (e.g., *Drosophila forficata*) and Hemiptera (*Nesophrosyne craterigena*), which they propose is explained by a reliance of both Drosophila and Nesophrosyne species on shared host plants across their ranges. Together, plant utilization and feeding habits may help explain why most native Drosophilidae species were infected with *Wolbachia* from supergroup B, why some members were infected with supergroup A (modified mouthparts group), and why identical alleles were shared between some distantly related taxa. Our findings are thus congruent with Bennet et al. [2], who proposed that horizontal transmission of *Wolbachia* occurs between Hawaiian taxa at multiple taxonomic scales.

Insects that possess piercing-sucking mouthparts may be more apt to transmitting *Wolbachia* to plants through feeding [19,54], and *Wolbachia* has been found to exist within insect salivary glands in addition to other somatic tissues [24,55]. Additionally, honeydew and infected leaves have been implicated in previous studies as a potential means of horizontal transmission [25,56]. Most non-native *Drosophila* included in this study were infected with supergroup A; however, infection by supergroup B *Wolbachia* within non-native *D. suzukii* individuals could be explained by their occasional use of native plants [31]. Full strain typing profiles, if available, could be used to test this idea. In other biological systems, although extremely rare, *Wolbachia* strains have been known to rapidly displace other strains, often in association with insect invasions. For example, the *Wolbachia* variant *w*Ri rapidly displaced *w*Au within their host *D. simulans* [57], and horizontal transmission occurred for *Wolbachia* endosymbionts and their host silverleaf whitefly (*Bemisia tabaci*), in which a host shift event occurred in China from indigenous members of the complex to the invader as well as from the invader to indigenous relatives [24]. An alternative explanation to plant-mediated horizontal transfer of *Wolbachia* is through non-lethal probing of infected nymphs and uninfected nymphs by parasitoid wasps ([24], reviewed by Sanaei et al. [58]). That mechanism for transmission is consistent with Bennett and colleagues [2] who postulated parasitoids to be a potential mechanism of horizontal transmission for *Wolbachia* in Hawaiian taxa, in addition to plant associations. They found that parasitoids, along with native and non-native Drosophila species, were grouped closely together based on the phylogenetic reconstruction of the *wsp* gene.

### 4.2. Discrepancy in Supergroup Designation of Loci

Whether supergroups can recombine has been the subject of debate. Ellegaard et al. [38] proposed that *Wolbachia* supergroups are irreversibly separated, and that barriers other than host-specialization are able to maintain distinct clades in recombining endosymbiont populations. Their conclusion was based on naturally occurring double-infections of *Wolbachia* strains *w*Ha and *w*No endosymbionts of *D. simulans*. Recent findings from a survey of 33 genome-sequences for *Wolbachia* strains belonging to supergroups A–F found that strains maintained a supergroup relationship across 210 conserved single-copy genes, yet an analysis of interclade recombination screening revealed that 14 inter-supergroup recombination events had occurred in six of the 210 core genes (6/210 = 2.9%) [59]. Consistent with recombination events, Baldo et al. [60]) found evidence for recombination between *gatB* and *fbpA* alleles, and intragenic re-combination was detected by comparing patterns of *gltA* to other housekeeping genes [60]. In this study, among the 44 *Wolbachia* strains isolated from Hawaiian Drosophilidae hosts, conflicting supergroup designations were observed for 20.4% of the strains (with data available at two or more genes), which in some cases resulted in an intermediate phylogenetic placement between supergroups A and B. In particular, *coxA* and *hcpA* alleles exhibited discordance between supergroup placement, congruent with discordance in supergroup designation for *coxA* and *hcpA* alleles observed within Lepidoptera species collected from West Siberia [61]. Although we cannot fully rule out that allelic discordance across strains may be a result of preferential amplification of certain alleles by primers in the presence of multiple infections—for example, double infections by strains belonging to supergroups A and B were observed to occur within w208 *D. apodasta* and w215 *D. nr. perissopoda*—the majority of individuals with conflicting alleles lacked evidence for the presence of a double infection. Therefore, the discrepancy in supergroup assignment between alleles may have resulted from a recombination event that occurred within a doubly infected host species and subsequent fixation of alleles. Further research could help to elucidate the complex interactions of endosymbionts and host taxa occurring within Hawaiian insect communities.

### 4.3. Conservation Implications

The rapid diversification of Hawaiian *Drosophila* results from a combination of evolutionary-time scale island isolation, rugged topography, and development of novel host plant associations that have persisted for millions of years [3]. Many species are single-island endemics with narrow ranges and are restricted to the natural distribution of their host plants, which makes populations especially vulnerable habitat degradation and climate change. At present the US Fish and Wildlife Service lists 13 Hawaiian Drosophilds as endangered (*D. aglaia*, *D. differens*, *D. digressa*, *D. hemipeza*, *D. heteroneura*, *D. montgomeryi*, *D. mulli*, *D. musaphilia*, *D. neoclavisetae*, *D. obatai*, *D. ochrobasis*, *D. sharpi*, *D. substenoptera*, and *D. tarphytrichia*) and one as threatened (*D. musaphilia*). These listed species represent 14.4% of all insects, and 4.8% of all listed invertebrates, within the USA (ECOS Environmental Conservation Online System https://ecos.fws.gov/ecp, accessed on 5 March 2023). Given *Wolbachia*’s impacts on reproduction, consideration of host–symbiont relationships and infection status might increase success of breeding programs and ensure that translocation efforts do not suffer from effects of cytoplasmic incompatibility. With regards to climate change, experimental data for Hawaiian *Drosophila* has demonstrated that species are locally adapted [62,63], thus, resilience to warming temperatures could perhaps be enhanced by manipulation of the host microbiomes, including *Wolbachia* endosymbionts. Endosymbiont-mediated responses to temperature stress are known to include transcription response and behavior [64,65].

## 5. Conclusions

This study sheds light on the infection status and coevolutionary history of *Wolbachia* endosymbionts within their Hawaiian Drosophilidae hosts. Co-phylogenetic reconciliations and comparative phylogenetic analyses indicate that the transmission patterns of *Wolbachia* is best explained by both co-speciation and host-switching events. Future studies that survey *Wolbachia* from a greater breadth of native Hawaiian arthropod taxa, as well as introduced arthropod invasive taxa, may help to improve our understanding of how *Wolbachia* transmission has occurred in Hawaiian ecosystems. Insights into *Wolbachia* infections and strain types could help guide conservation programs, possibly enhancing translocation efforts, impacting host behavioral response to temperatures, and conferring host thermal tolerance.

## Figures and Tables

**Table 1:** Data for *Wolbachia* genetic sequences used for the purpose (Purpose) of in silico extraction of sequence from genomes for primer redesign (PR) or *Wolbachia* allele strain typing and/or phylogenetic analysis (A/P). Shown are *Wolbachia* host species names, *Wolbachia* strain abbreviations, host collection locations or laboratory sources if known, National Center for Biotechnology Information (NCBI) accessions, genome references, and *Wolbachia* supergroup designations.

*Wolbachia* Host	*Wolbachia* Strain	Host Collect Location	Genome Accession	Citation	Supergroup	Purpose
*Drosophila recens*	*w*Rec	Rochester, New York, USA	GCF_000742435.1	[37]	A	PR
*D. melanogaster*	*w*Mel	Stock Center *D. melanogaster* strain yw67c23	GCF_000008025.1	[35]	A	PR, A/P
*D. simulans*	*w*No	Noumea, New Caledonia	GCF_000376585.1	[38]	B	PR, A/P
*D. simulans*	*w*Ha	Hawai’i, USA	GCF_000376605.1	[38]	A	A/P
*D. ananassae*	*w*Ana	Tucson Strain Center [strain 14024-0371.13]	GCF_000167475.1	[39]	A	PR
*D. suzukii*	*w*Suzi, strain valsugana	Trentino Alto Adige, Italy	GCF_000333795.1	[40]	A	PR, A/P
*Culex quinquefasciatus*	*w*Pip strain Pel	Sri Lanka	AM999887.1	[41]	B	A/P
*C. quinquefasciatus*	*w*Pip strain JHB	Johannesburg, Africa	ABZA0100000	[42]	B	A/P
*Aedes albopictus*	*w*AlbA	Unknown	^1^	[34]	A	A/P
*Brugia malayi*	*w*Bm	TRS Lab colony (Athens, GA, USA)	NC_006833.1	[43]	D	A/P
*Cimex lectularius strain* JESC	*w*Cle	Japan	AP013028.1	[44]	F	A/P

^1^ “isolate 12”, typed using MLST by Baldo et al. [34], NCBI accessions DQ842268.1, DQ842342.1, DQ842379.1, DQ842305.1.

**Table 2:** A comparison of numbers of individual Hawaiian Drosophildae (genus *Drosophila*) species with at least five specimens per species screened, per-species total numbers of individuals screened, number of individuals with confirmed *Wolbachia* infections, numbers of individuals having no confirmed infections but positive for PCR amplifications that failed sequencing, total number of individuals having zero amplifications across all loci, and the proportion of infected individuals by species.

Species	Screened	Confirmed Infected	PCR Product Only	Zero Amplifications	Proportion Infected
*D. ciliaticrus*	5	0	0	5	0.00
*D. engyochracea*	7	2	2	3	0.29
*D. hawaiiensis*	15	2	0	13	0.13
*D. heteroneura*	5	2	0	3	0.40
*D. murphyi*	5	2	0	3	0.40
*D. ochracea*	11	1	1	9	0.09
*D. odontophallus*	5	0	0	5	0.00
*D. orphnopeza*	7	0	1	6	0.00
*D. primaeva*	11	0	1	10	0.00
*D. silvestris*	6	0	0	6	0.00
*D. sproati*	114	0	5	109	0.00
*D. tanythrix*	10	0	0	10	0.00
*D. yooni*	10	2	1	7	0.20
totals	219	17	11	191	n/a

n/a = not applicable.

**Table 3:** A list of Hawaiian Drosophilidae, invasive mosquito, and outgroup host species screened for *Wolbachia* infections using PCR amplification and verified by Sanger sequencing. The five gene targets were amplified using a modified version of the multi-locus strain typing (MLST) approach for strain assignment to supergroup (see text for details). For each gene, alleles were assigned to a supergroup based on single-gene phylogenetic reconstructions, and unique sequences were assigned an arbitrary allele number. In some cases, supergroup assignments were discordant across alleles, and alleles that could not be assigned to a supergroup are denoted as (?). *Wolbachia* endosymbionts of double-infected hosts are denoted by bold font. MLST genes that failed amplification and/or sequencing are denoted as ‘---’.

	*Wolbachia* Sample Name	Host Species Name	Island of Collection	*coxA*	*fbpA*	*hcpA*	*ftsZ*	*gatB*	Strain Type
Native Hawaiian Drosophilidae	247w	*Drosophila engyochracea*	Hawai‘i	**2 (A)**	**---**	**11 (B)**	**2 (A)**	**---**	A/B
	**250_1w**	*D. engyochracea*	Hawai‘i	**2**	**---**	**---**	**2**	**---**	**A**
	**250_2w**	*D. engyochracea*	Hawai‘i	**5**	**---**	**---**	**10**	**---**	**A**
	264w	*D. hawaiiensis*	Hawai‘i	**2**	**---**	**---**	**---**	**---**	A
	266w	*D. hawaiiensis*	Hawai‘i	**2 (A)**	**---**	**11 (B)**	**2 (A)**	**---**	A/B
	252w	*D. heteroneura*	Hawai‘i	**2 (A)**	**---**	**11 (B)**	**---**	**---**	A/B
	253w	*D. heteroneura*	Hawai‘i	**6 (A)**	**---**	**11 (B)**	**---**	**---**	A/B
	**16_1w**	***D. large spots***	Hawai‘i	**2**	**7**	**6**	**2**	**---**	**A**
	**16_2w**	***D. large spots***	Hawai‘i	**4**	**8**	**2**	**1**	**---**	**B**
	171w	*D. murphyi*	Hawai‘i	**---**	**4 (A)**	**3 (?)**	**4 (B)**	**---**	A/?/B
	MLL6w (415)	*D. murphyi*	Hawai‘i	**13**	**---**	**1**	**---**	**---**	B
	244w	*D. nigrocirrus*	Hawai‘i	**2**	**6**	**5**	**11**	**---**	A
	256w	*D. ochracea*	Hawai‘i	**2 (A)**	**3**	**11 (B)**	**---**	**---**	A/B
	123w	*D. prolaticilia*	Hawai‘i	**1**	**1**	**2**	**1**	**3**	B
	197w	*D. prolaticilia*	Hawai‘i	**1**	**1**	**2**	**1**	**---**	B
	221w	*D. seclusa*	Hawai‘i	**1**	**1**	**1**	**6**	**1**	B
	291w	*D. yooni*	Hawai‘i	**13**	**---**	**---**	**---**	**---**	B
	292w	*D. yooni*	Hawai‘i	**13**	**---**	**---**	**---**	**---**	B
	20w	*Scaptomyza caliginosa*	Hawai‘i	**1**	**1**	**1**	**6**	**1**	B
	152w	*S. cyrtandrae*	Hawai‘i	**2 (A)**	**---**	**11 (B)**	**---**	**---**	A/B
	204w	*S. reducta*	Hawai‘i	**---**	**---**	**11**	**---**	**---**	B
	205w	*S. reducta*	Hawai‘i	**3**	**2**	**11**	**---**	**---**	B
	206w	*S. undulata*	Hawai‘i	**1**	**---**	**14**	**---**	**---**	B
	185w	*D. ancyla*	Maui	**13**	**12**	**1**	**7**	**1**	B
	175w	*D. prostopalpis*	Maui	**1**	**2**	**2**	**1**	**4**	B
	145w	*D. quasiexpansa*	Maui	**1**	**---**	**4**	**4**	**---**	B
	216w	*D.* nr. *redunca*	Hawai‘i	**1**	**1**	**1**	**9**	**---**	B
	200w	*S. crassifemur*	Maui	**1**	**---**	**1**	**---**	**---**	B
	201w	*S. crassifemur*	Maui	**9**	**10**	**11**	**---**	**---**	B
	202w	*S. nasalis*	Maui	**1**	**---**	**---**	**---**	**---**	B
	203w	*S. nasalis*	Maui	1	3	1	**---**	**---**	B
	**208_1w**	***D. apodasta***	Kaua‘i	**8 (A)**	**---**	**11 (B)**	**---**	**---**	**A/B**
	**208_2w**	***D. apodasta***	Kaua‘i	**3**	**---**	**11**	**---**	**---**	**B**
	187w	*D. atroscutellata*	Kaua‘i	**13**	**13**	**1**	**4**	**---**	B
	41w	*D.* nr. *basimacula #2*	Kaua‘i	**13**	**1**	**1**	**5**	**1**	B
	59w	*D.* nr. *basimacula #1*	Kaua‘i	**13**	**1**	**1**	**3**	**---**	B
	209w	*D. basimacula*	Kaua‘i	**1**	**---**	**11**	**---**	**---**	B
	212w	*D.* nr. *basimacula #1*	Kaua‘i	**13**	**1**	**1**	**3**	**1**	B
	213w	*D.* nr. *basimacula #2*	Kaua‘i	**13**	**1**	**1**	**5**	**---**	B
	5w	*D.* nr. *basimacula #5*	Kaua‘i	**13**	**1**	**1**	**3**	**1**	B
	127w	*D. kikiko*	Kaua‘i	**7**	**---**	**8**	**---**	**---**	B
	155w	*D. micromyia*	Kaua‘i	**10**	**11**	**7**	**8**	**2**	B
	**215_1w**	***D.* nr. *perissopoda #1***	Kaua‘i	**1 (B)**	**5 (A)**	**---**	**---**	**---**	**A/B**
	**215_2w**	***D.* nr. *perissopoda #1***	Kaua‘i	**13**	**9**	**---**	**---**	**---**	**B**
**Non-native Drosophila and mosquitoes collected in Hawaii**	3_A12w	*D. suzukii*	Kaua‘i	**---**	**---**	**12**	**---**	**---**	A
	3_B11w	*D. suzukii*	Kaua‘i	**---**	**---**	**14**	**---**	**---**	B
	3_C2w	*D. suzukii*	O‘ahu	**15**	**17**	**14**	**---**	**---**	B
	3_C3w	*D. suzukii*	Kaua‘i	**16**	**---**	**13**	**---**	**---**	A
	3_D5w	*D. suzukii*	Kaua‘i	**---**	**---**	**13**	**---**	**---**	A
	3_E3w	*D. suzukii*	Kaua‘i	**---**	**---**	**13**	**---**	**---**	A
	3_F6w	*D. suzukii*	Kaua‘i	**---**	**---**	**14**	**---**	**---**	B
	3_H4w	*D. suzukii*	Kaua‘i	**---**	**---**	**13**	**---**	**---**	A
	wHa ^6^	*D. simulans wHa*	Hawai‘i	**16**	**20**	**13**	**15**	**---**	A
	wAlb	*Aedes albopictus*	Hawai‘i	**11 (A)**	**14 (B)**	**9 (B)**	**12 (B)**	**---**	A/B
	6771w	*Culex quinquefasciatus*	Hawai‘i	**13**	**16**	**11**	**14**	**---**	B
**Other Drosophila and Mosquitoes**	wAlbA ^1^	*A. albopictus*	Unknown	**12**	**15**	**10**	**13**	**---**	A
	*w*Pip ^2^	*C. quinquefasciatus Pel*	Sri Lanka	**13**	**16**	**11**	**14**	**---**	B
	*w*Pip ^3^	*C. quinquefasciatus JHB*	Johannesburg	**13**	**16**	**11**	**14**	**---**	B
	*w*Dmel ^4^	*D. melanogaster*	Laboratory Stock	**17**	**19**	**15**	**16**	**---**	A
	*w*Dsuzi ^5^	*D. suzukii*	Italy	**14**	**18**	**12**	**15**	**---**	A
	wNo ^6^	*D. simulans*	New Caledonia	**18**	**21**	**16**	**17**	**---**	B
**O G**	wBm ^7^	*Brugia malayi*	Unknown	**19**	**22**	**17**	**18**	**5**	D
	wCle ^8^	*Cimex lectularius*	Unknown	**20**	**23**	**18**	**19**	**6**	F

^1^ “isolate 12”, typed using MLST by Baldo et al. [34]; ^2^ genome accession AM999887.1, Klasson et al. [41]; ^3^ genome accession ABZA01000002.1, Salzberg et al. [42]; ^4^ genome accession NC_002978.6, Wu et al. [35]; ^5^ *Wolbachia* endosymbiont str. *valsugana*, WGS project CAOU00000000, Siozios et al. [40]; ^6^ genome accession NC_021084.1, Ellegaard et al. [38]; ^7^ Wolbachia endosymbiont strain TRS, NC_006833.1, Foster et al. [43]; ^8^ Nikoh et al. [44].

**Table 4:** Co-phylogenetic reconstructions implemented in JANE4 (see text for details) with cost-scheme parameters loss, failure to diverge, and duplication each set to 1, duplication and host switch set to 2, and varying the co-speciation cost (Cost) by 0 or 1.

	*Drosophila* and *Wolbachia*		Invasive Mosquitoes, *Drosophila*, and *Wolbachia*	
	Cost 0	Cost 1	Cost 0	Cost 1
Co-speciation	8	8	9	7
Duplication	0	0	0	1
Duplication and host switches	7	7	8	9
Loss	1	1	2	1

## Data Availability

The *Wolbachia* sequence data generated for this study (primer re-design and data analysis) are available from Supplementary Datafile S1; Drosophilidae sequences are available from the National Center for Biotechnology Information public repository.

## Supplementary Materials

The following supporting information can be downloaded at https://www.mdpi.com/article/10.3390/genes14081545/s1, Table S1. National Center for Biotechnology Information (NCBI) accession numbers for Hawaiian Drosophilidae gene sequences selected for phylogenetic reconstruction of individual species having a verified *Wolbachia* infection, and genome accessions for outgroup taxa (also infected) [3,66,67,68,69,70]. Table S2. Records for amplification and sequencing of *Wolbachia* endosymbionts of Hawaiian Drosophilids. Table S3. Record of Akaike information criterion (AIC) values obtained using the fitMK function in phytools v0.6-44 (Revell 2012) [51] package in R to determine the best rates model to apply to each data set for stochastic character mapping analyses. Figure S1. Phylogenetic reconstruction of *Wolbachia* housekeeping genes based on Bayesian inference analyses (see main text for details): (a) cytochrome C oxidase subunit A (*coxA*) [378 bp], with 46 sequences, (b) conserved hypothetical protein (*hcpA*) [381 bp], with 44 sequences; (c) fructose-bisphosphate aldolase (*fbpA*) [417 bp], with 30 sequences, and (d)) cell division protein (*ftsZ*) [354 bp], with 28 sequences. Individuals consistent in their supergroup designations across all genes considered are indicated as either pink for supergroup A or purple for supergroup B. Individuals that showed conflicting supergroup designation between genes are shown in grey. Outgroup taxa belonging to supergroups D and F are shown in green. A solid line indicates that supergroup designation was based on three or more genes, whereas a dotted line indicates that data for 2 of fewer genes were available for super group designation. The taxonomic standing is uncertain for *Wolbachia* endosymbiont host species *Drosophila basimacula* #5 and #2 (samples 5 and 41), *D. quasiexpansa* sample 145, *D. redunca* sample 216 and *D. perrisopoda* sample 215 (see main text for details). Figure S2. Phylogenetic reconstruction of *Wolbachia* housekeeping genes based on Maximum Likelihood analyses (see main text for details]): (a) cytochrome C oxidase subunit A (*coxA*) [378 bp], with 46 sequences, (b) conserved hypothetical protein (*hcpA*) [381 bp], with 44 sequences; (c) fructose-bisphosphate aldolase (*fbpA*) [417 bp], with 30 sequences, and (d) cell division protein (*ftsZ*) [354 bp], with 28 sequences. Individuals consistent in their supergroup designations across all genes considered are indicated as either pink for supergroup A or purple for supergroup B. Individuals that showed conflicting supergroup designation between genes are shown in grey. Outgroup taxa belonging to supergroups D and F are shown in green. A solid line indicates that supergroup designation was based on three or more genes, whereas a dotted line indicates that data for two or fewer genes were available for super group designation. The taxonomic standing of *Wolbachia* endosymbiont host species *Drosophila basimacula* #5 and #2 (samples 5 and 41), *D. quasiexpansa* sample 145, *D. redunca* sample 216 and *D. perrisopoda* sample 215 is uncertain (see main text for details). Figure S3. Phylogenetic reconstruction of three concatenated *Wolbachia* MLST genes: cytochrome C oxidase subunit A (*coxA*)*,* conserved hypothetical protein (*hcpA*), and cell division protein (*ftsZ*) [1113 bp] based on a by gene and codon partitioning scheme and 25 sequences analyzed using (A) Bayesian and (B) Maximum Likelihood approaches. Individuals consistent in their supergroup designation across all available MLST gene data are shown pink for supergroup A or purple for supergroup B. Individuals that showed conflicting supergroup designation between genes are shown in grey. Outgroup taxa (Supergroups D and F) are shown in green. The taxonomic standing of *Wolbachia* endosymbiont host species *Drosophila basimacula* #5 and #2 (samples 5 and 41), *D. quasiexpansa* sample 145, and *D. redunca* sample 216 is uncertain (see main text for details). Figure S4. Phylogenetic reconstruction of four concatenated *Wolbachia* MLST genes: cytochrome C oxidase subunit A (*coxA*)*,* fructose-bisphosphate aldolase (*fbpA*), conserved hypothetical protein (*hcpA*), and cell division protein (*ftsZ*) [1530 bp] based on a by gene and codon partitioning scheme and (A) Bayesian and (B) Maximum Likelihood approaches. Individuals consistent in their supergroup designation across all available MLST gene data are shown pink for supergroup A or purple for supergroup B. Individuals that showed conflicting supergroup designation between genes are shown in grey. Outgroup taxa (Supergroups D and F) are shown in green. The taxonomic standing of *Wolbachia* endosymbiont host species *Drosophila basimacula* samples 5 and 41 and *D. redunca* sample 216 are uncertain (see main text for details). Figure S5. Phylogenetic reconstruction of Hawaiian Drosophilidae host species (*Drosophila* and *Scaptomyza*) and invasive mosquitoes *A. albopictus* and *C. quinquefasciatus* based on four concatenated genes, elongation factor 1-γ (*EF1g*), glycerol-3-phosphate dehydrogenase (*Gpdh*), phosphoglucose isomerase (*Pgi*), and yolk protein 2 (*Yp2*) [1812 bp] using a by codon partitioning scheme and Bayesian inference (A) or Maximum Likelihood (B). Phylogenetic analysis was restricted to Drosophilidae host species having *Wolbachia* sequence data available across three multilocus sequence typing genes (see main text for details). Figure S6. Implemented in JANE4 (Conow et al. 2010) [49], a possible solution for co-phylogenetic reconciliation analysis for Hawaiian Drosophilidae and their *Wolbachia* endosymbionts based on the cost scheme setting co-speciation assigned 0 (panel A) or 1 (panel B), with loss, failure to diverge, and duplication each set to 1 and duplication and host switch set to 2. Estimated biological events that best describe the data are depicted on the phylogeny [open circle: co-speciation; closed circle: duplication; closed circle with arrow: duplication and host switch; dashed line: loss]. Red indicates that the event is optimally placed, whereas yellow indicates that another placement exists that is equally valid. The taxonomic standings of *Wolbachia* endosymbiont host species *Drosophila basimacula* #5 and #2 (samples 5 and 41) and *D. perrisopoda* #1 (sample 215) are uncertain (see main text for details). Figure S7. Stochastic character mapping of the Hawaiian Drosophilidae host species’ ancestral traits (A) island of collection and (B) host plant families mapped to an unrooted *Wolbachia* phylogeny. External nodes depict host trait assignments: solid = known, equal proportions = unknown. Interior nodes represent posterior probabilities that the host’s character trait is ancestral and congruent with the phylogenetic signal of the *Wolbachia* strain. Key to *Wolbachia* found in host individual listed in Supplementary S1 (sample number, species name): (1) 244 w *D. nigrocirrus*, (2) 16_1w *D. “*large spots” (double-infected strain A), (3) 185w *D. ancyla*, (4) 221w *D. seclusa*, (5) 20w *S. caliginosa*, (6) 216w *D.* nr. *redunca*, (7) 175w *D. prostopalpis*, (8) 123w *D. prolaticilia,* (9) 16_2w *D. “*large spots” (double-infected strain B, (10) 5w *D.* nr. *basimacula #5*, (11) 187w *D. atroscutellata*, (12) 145 w *D. quasiexpansa*, (13) 41w *D.* nr. *basimacula #2*, (14) 155 w *D. micromyia,* (15) 266w *D. hawaiiensis*, and (16) 247w *D. engyochracea*. Two strains belong to supergroup A, two were intermediate A/B, and all other strains belong to supergroup B. Supplementary Information. Extended methods [34,35,71,72,73,74,75,76]. Supplementary Datafile S1. *Wolbachia* sequence data generated for this study and used for primer re-design and data analyses. Supplementary S1. Hawaiian Drosophilidae specimens (*n* = 399, genus *Drosophila* and *Scaptomyza*) and invasive *Drosophila* (*n* = 68) screened for the presence of endosymbiotic bacteria *Wolbachia* [3,32]. Supplementary S2. A list of *Wolbachia* host species and sequence accessions for five *Wolbachia* Multiple Locus Strain Typing sequences [cytochrome C oxidase subunit A (*coxA*), conserved hypothetical protein (*hcpA*), fructose-bisphophate adolase (*fbpA*), cell division protein (*ftsZ*) and aspartyl/glutamyl-tRNA aminotransferase subunit B (*gatB*)], and Wolbachia surface protein [*wsp* and paralog *wspB*] used for BLASTn queries as part of primer re-design efforts.
