# Supplementary material for: The Transmission Patterns of the Endosymbiont Wolbachia within the Hawaiian Drosophilidae Adaptive Radiation
Source: Genes (Basel). 2023 Jul 27;14(8):1545. doi: 10.3390/genes14081545 (PMC10454618; doi:10.3390/genes14081545)
Supplement: Supplementary file 1 [file genes-14-01545-s001.zip › Supplementary Information - Extended Methods.pdf]

genes

MDPI

Supplementary Information for Manuscript: The transmission patterns of the endosymbiont *Wolbachia* within the Hawaiian Drosophilidae adaptive radiation

I. Extended Methods

Primer re-design

We redesigned and tested primers for the multilocus gene panel (MLST) by extracting sequence data from five *Wolbachia* genomes using gene specific searches as queries (see main text). Each genome was converted to a nucleotide database to facilitate BLASTn (v 2.2.30) searches for target sequences using MLST reference sequences as queries (see main text), plus the initial set of sequences produced as part of this study. After BLASTn filtering of hits using a threshold e-value < 0.001 the hit regions were *in silico* excised from each genome by cutting the target genomic interval with an excess of 200 base pairs on both the 5′ and 3′ ends, aligned, and then primers were manually re-designed following generally accepted primer design criteria: 1) GC clamp at 3′ end, 2) 18-21 nucleotides in length, 3) no homopolymer runs greater than 5 nucleotides, and 4) ~50% GC content.

Table 1. Multilocus sequence technique (MLST) primers (with citations) and re-designed primers used in this study, plus approximate amplicon lengths in base pairs (bp). Data shown only for primer combinations that produced additional amplicons.

	Primer Name	Sequence (5′-3′)	Citation	Product Size (bp)
Previously-Designed Primers	MLST-gatB_F1	GAKTTAAAYCGYGCAGGBGTT	[1]	369
	MLST-gatB_R1	TGGYAAYTCRGGYAAAGATGA	[1]	
	MLST-coxA_F1	TTGGRGCRATYAACTTTATAG	[1]	402
	MLST-coxA_R1	CTAAAGACTTTKACRCCAGT	[1]	
	MLST-hcpA_F1	GAAATARCAGTTGCTGCAAA	[1]	444
	MLST-hcpA_R1	GAAAGTYRAGCAAGYTCTG	[1]	
	MLST-ftsZ_F1	ATYATGGARCATATAAARGA-TAG	[1]	435
	MLST-ftsZ_R1	TCRAGYAATGGATTRGATAT	[1]	
	MLST-fbpA_F1	GCTGCTCCRCTTGGYWTGAT	[1]	429
	MLST-fbpA_R1	CCRCCAGARAAAAYYACTATTC	[1]	
	MLST-wsp_F1	GTCCAATARSTGATGARGAAAC	[1]	546
	MLST-wsp_R1	CYGCACCAAYAGYRCTRTAAA	[1]	
	wspB_F	TTTGCAAGTGAAACAGAAGG	[2]	786 – 822
	wspB_R	GCTTTGCTGGCAAAATGG	[2]	
Re-De-	gatB_Forward	TTAAATCGTGCAGGSGTTGC	This Study	469
	MLST-gatB_R1	TGGYAAYTCRGGYAAAGATGA	[1]	
	coxA_Forward1	GGTATGTCRTCAATTGTTGGG	This Study	504
	MLST-coxA_R1	CTAAAGACTTTKACRCCAGT	[1]	

	coxA_Forward1	GGTATGTCRTCAATTGTTGGG	This Study	372
	coxA_Reverse	CCTATCATDRCATAAAC-CATYCC	This Study	
	hcpA_Forward1	CAGYTGCTGCAAARCAAGGG	This Study	501
	hcpA_Reverse	GAGCAAGTTCTGGTTCTCC	This Study	
	ftsZ_Forward1	TGGTGCTTTGCCTGATGTTGG	This Study	529
	ftsZ_Reverse1	ATCTTCTCCTTCTGCCTCTCC	This Study	
	ftsZ_Forward2	ATTACCGTTGTGGGAGTGG	This Study	725
	ftsZ_Reverse2	GCYTCTGCAGCACTAATTGC	This Study	
	fbpA_Forward	AGCTGGTGCTKCAACTTATGC	This Study	486
	MLST-fbpA_R1	CCRCCAGARAAAAYYACTATTC	[1]	
	wsp_Forward1	CGTTTRCAATAYAAYGGTG	This Study	467-473
	wsp_Reverse1	CCAWAAGARCCRAARTAACG	This Study	
	wspB_F	TTTGCAAGTGAAACAGAAGG	[2]	764 – 800
	wspB_Reverse1	GTAAGACCAGCYTCTATRCC	This Study	

PCR cycling conditions and DNA sequencing

The MLST gene targets and *wsp* genes were amplified with polymerase chain reaction (PCR) as follows. Each gene was amplified in a 20-µl reaction containing 1X PCR buffer, 1.8 mM of $MgCl_2$, 0.225 µM of each primer, 0.155 mM of each deoxynucleoside triphosphate, 0.025 U *Taq* DNA polymerase, 1.5-µl template DNA, 1.0-µl Tween 20, and 0.4-µl DMSO. Reactions were incubated at 94°C for 2 min to initiate the amplification process, followed by 35 cycles of 94°C for 30 sec, 45 sec at the determined optimal annealing temperature of each set of primers, and 90 sec at 72°C, with a final extension step of 10 min at 70°C. For published "original" primers, the annealing temperatures were: 54°C: *gatB* and *ftsZ;* 55°C: *hcpA, coxA and wspB;* and 59°C: *fbpA and wsp*). The most effective combinations of re-designed and previously designed primers were found to have the following annealing temperatures: 54 °C: *wsp* and *wspB*; 55 °C: *gatB* and *coxA;* 57°C: *hcpA* and *fbpA;* and 59°C: *ftsZ*). All PCRs were performed using an Applied Biosystems ProFlex thermocycler and PCR products were visualized using 1.5% agarose gel and Gel Red.

Following amplification, PCR products were purified based on the number and types of bands present. Samples with a single, distinct band were enzymatically cleaned with EXOSAP (Invitrogen) per manufacturer's protocol. Products that showed either the presence of multiple bands or large amounts of primer dimers were cleaned with an Invitrogen 2% Size-Select E-gel stained with SyberGold following manufacturer's instructions. However, in place of nuclease-free water, all recovery wells were filled with 50-µl of TE buffer (10mM Tris and 0.1mM EDTA) as it allowed for better storage and preservation of the collected PCR product. Following PCR clean-up, samples were prepped and submitted for Sanger sequencing at the University of Hawaii at Hilo's Evolutionary Genomics Core Facility. Chromatograms of sequences were then viewed and edited using Sequencher version 5.2.4 (Gene Codes Corporation).

Extended Methods, Phylogenetics

Phylogenetic reconstructions of *Wolbachia* based on concatenated gene trees using MLST gene datasets with five, four, and three genes were used to evaluate the impact of incomplete (and modified MLST) sequence data sets on tree topology and support. These data sets consisted of five [5-genes, 1980 bp], four [4-genes, 1530 bp, minus *gatB*], and three [3-genes, 1113 bp; minus *gatB* and *fbpA*] concatenated gene sequences. We were only able to generate a five-gene MLST profile for nine *Wolbachia* strains, all of which belonged to supergroup B and were clearly differentiated from outgroup taxa D and F. Across the four- and three-gene trees (Figure 1, Panels A and B) members of supergroups A and B consistently grouped into their respective clades, although some individuals exhibited intermediate supergroup assignments, consistent with our observation of conflicting gene assignments in the single-gene trees (see main text for details).

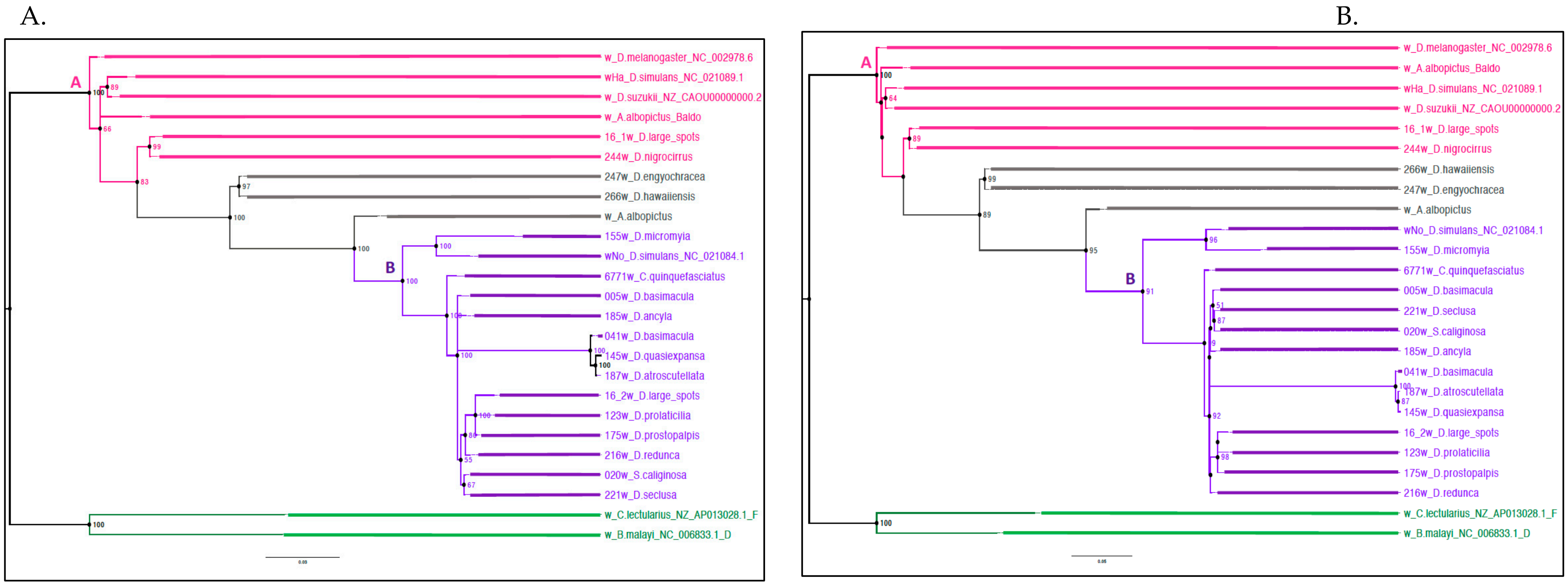

Figure 1. Phylogenetic reconstruction of three concatenated Wolbachia MLST genes: *coxA*, *hcpA*, and *ftsZ* [1113 bp] based on a by gene and codon partitioning scheme and 25 sequences analyzed using (A) Bayesian and (B) Maximum Likelihood approaches. Individuals consistent in their supergroup designation across all available MLST gene data are shown pink for supergroup A or purple for supergroup B. Individuals that showed conflicting supergroup designation between genes are shown in grey. Outgroup taxa (Supergroups D and F) are shown in green. The taxonomic standing is uncertain for Drosophilidae host samples 145_*D. quasiexpansa*, 5_*D. basimacula* #5, 41_*D. basimacula* #2, and 216_*D. redunca*.

Justification for phylogenetic analysis method (Wolbachia)

For Bayesian inference, model selection in MrBayes (v3.2.5; [3]) was evaluated based on: 1) the best partitioning scheme (i.e., no partition, codon [position specific], gene, or gene + codon position) and 2) the best nucleotide substitution model (i.e., GTR + G or sampling across total GTR model space + G). A stepping-stone sampling analysis was applied to estimate the marginal likelihoods of the models and assess the "best model", as opposed to the more simplistic harmonic mean method, which has been determined to be far less accurate and precise [4,5,6]). The stepping stone sampling analyses were run for 5 million generations and run diagnostics were computed and printed every 25,000 generations. The best model was determined as having the greatest marginal likelihood value, with a difference in model likelihood values greater than 3 on a logarithmic scale taken as strong support in favor of the best model [6,7] (Table 2). Based on stepping stone analyses, the nucleotide substitution model that consistently had the greatest marginal likelihood for both *Wolbachia* and host species' concatenated data sets was GTR + G. The partition scheme, on the other hand, showed mixed evidence for either "codon position" or "gene + codon position" as being the best model, however in most cases there was no significant statistical difference detected between the likelihood scores. Therefore, analyses for the host species' and *Wolbachia* data sets were run using both by "codon position" and "gene + codon position" partitioning schemes, respectively.

The partitioning scheme "codon" or "gene and codon" and analysis method, Bayesian versus Maximum-Likelihood, had little effect on tree topologies (Main text Figures S1-S4). Although slight differences in support values between Bayesian and ML analyses occurred, *i.e.*, posterior probabilities tended to be a greater than bootstrap values, that is expected for those two types of analysis, and trees can still be considered congruent [8].

Table 2. Record of marginal likelihoods obtained from a stepping stone analysis performed in MrBayes v3.2.5 [3] to tes[illegible] scheme and evolutionary rate model to be applied for each data set for phylogenetic reconstruction analyses.

**Concatenated Nucleotide Data sets**	**Partitioning Scheme**				**Evolutionary [illegible]**		
	**No Partition**	**By Codon**	**By Gene**	**By Gene &Codon**	**GTR+gamma**	**Mixed+gamma**	**[illegible]**
***Wolbachia* 5 genes**	-6104.02	-5984.22	-5985.36	-5774.11	-5774.36	-5779.44	[illegible]
***Wolbachia* 4 of 5 genes**	-4100.34	-3957.57	-4092.6	-3956.6	-3956.73	-3958.68	[illegible]
***Wolbachia* 3 of 5 genes**	-2949.51	-2852.94	-2940.84	-2850.14	-2850.07	-2852.68	[illegible]
***Wolbachia* 4 genes**	-5690.12	-5537.27	-5686.08	-5543.92	-5543.97	-5547.3	[illegible]
***Wolbachia* 4 of 3 genes**	-4001.58	-3892.69	-4001.18	-3894.04	-3893.83	-3897.63	[illegible]
***Wolbachia* 3 genes**	-4170.27	-4074.49	-4171.32	-4077.5	-4077.35	-4081.24	[illegible]
**Host species**	-8543.47	-8262.23	-8548.51	-8262.42	-8262.1	-8266.03	[illegible]

Non-endorsement disclaimer: Any use of trade, firm, or product names is for descriptive purposes only and does not imply endorsement by the U.S. Government.

References

1. Baldo, L.; Dunning Hotopp, J.C.; Jolley, K.A.; Bordenstein, S.R.; Biber, S.A.; Choudhury, R.R.; Hayashi, C.; Maiden, M.C.J.; Tettelin, H.; Werren, J.H. Multilocus sequence typing system for the endosymbiont *Wolbachia pipienti*s. *Appl. Environ. Microbiol.* **2006**, *72*, 7098–7110.
2. Wu, M.; Sun, L.V.; Vamathevan, J.; Riegler, M.; Deboy, R.; Brownlie, J.C.; McGraw, E.A.; Martin, W.; Esser, C.; Ahmadinejad, N.; Wiegand, C. Phylogenomics of the reproductive parasite *Wolbachia pipientis w*Mel: A streamlined genome overrun by mobile genetic elements. *Public Libr. Sci. (PLOS) Biol.* **2004**, *2*, 327–341.
3. Ronquist, F.; Huelsenbeck, J.P. MrBayes3: Bayesian phylogenetic inference under mixed models. *Bioinformatics* **2003**, 19, 1572–1574
4. Ronquist, F.; Deans, A.R. Bayesian phylogenetics and its influence on insect systematics. Annu. Rev. Entomol. 2010, 55, 189–206.
5. Xie, W.; Lewis, P.O.; Fan, Y.; Kuo, L.; Chen, M.-H. Improving marginal likelihood estimation for Bayesian phylogenetic model selection. Syst. Biol. 2011, 60, 150–160.
6. Ronquist F.; Huelsenbeck J.; Teslenko, M. Draft MrBayes version 3.2 manual: Tutorials and model summaries. 2011. Available from: https://pbil.univ-lyon1.fr/members/perriere/pdf/mb3.2_manual.pdf
7. Kass, R.E.; Raftery, A.E. Bayes factors. J. Am. Stat. Assoc, 1995, 90, 773–795.
8. Huelsenbeck, J.; Rannala, B. Frequentist properties of Bayesian posterior probabilities of phylogenetic trees under simple and complex substitution models. Syst. Biol. 2004, 53904–53913.
