# Supplementary material for: The Transmission Patterns of the Endosymbiont Wolbachia within the Hawaiian Drosophilidae Adaptive Radiation
Source: Genes (Basel). 2023 Jul 27;14(8):1545. doi: 10.3390/genes14081545 (PMC10454618; doi:10.3390/genes14081545)
Supplement: Supplementary file 1 [file genes-14-01545-s001.zip › Supplementary_S2_Woblachia_NCBI_Accessions_06152023_07262023.pdf]

Appendix S2. A list of *Wolbachia* host species and sequence accessions for five *Wolbachia* Multiple Locus Strain Typing sequences [cytochrome C oxidase subunit A (*coxA*), conserved hypothetical protein (*hcpA*), fructose-bisphophate adolase (*fbpA*), cell division protein (*ftsZ*) and aspartyl/glutamyl-tRNA aminotransferase subunit B (*gatB*)], and *Wolbachia* surface protein [*wsp* and paralog *wspB*] used for BLASTn queries as part of primer re-design efforts. The sequences were downloaded from the National Center of Biotechnology Information (NCBI) GenBank repository.

coxA		***fbpA***		***hcpA***		***ftsZ***		***gatB***	
***Wolbachia* Host**	**Accession Number**	***Wolbachia* Host**	**Sample or Accession Number**	***Wolbachia* Host**	**Sample or Accession Number**	***Wolbachia* Host**	**Sample or Accession Number**	***Wolbachia* Host**	**Sample or Accession Number**
D. nr. *basimacula #5*	5 (this study)	*D. pandora* (MK)	KU686371.1	*D.* nr. *basimacula #5*	5 (this study)	*D.* nr. *basimacula #5*	5 (this study)	*D.* nr. *basimacula #5*	5 (this study)
S. calignosa	20 (this study)	*D. pandora* (wRi)	KU686365.1	*D.* nr. *basimacula* #1	59 (this study)	*D. simulans (wNo)*	DQ266426 .1	*S. calignosa*	20 (this study)
D. nr. *basimacula* #2	41 (this study)	*D. suzukii*	KS308223.1	*D. prolacticila*	123 (this study)	*D. innubila B*	DQ266424 .1	*D. prolacticila*	123 (this study)
D. nr. *basimacula* #1	59 (this study)			*D. micromyia*	155 (this study)	*D. simulans (Riverside)*	EF423735. 1	*D. micromyia*	155 (this study)
D. prolacticila	123 (this study)			*D. prostopalpis*	175 (this study)	*D. simulans (Irvine 8)*	EF423733. 1	*D. prostopalpis*	175 (this study)
D. kikiko	127 (this study)			*D. ancyla*	185 (this study)	*D. pandora (wRi)*	KU686366. 1	*D. ancyla*	185 (this study)
D. prostopalpis	175 (this study)			*D.* nr. *basimacula* #1	212 (this study)			*D.* nr. *basimacula* #1	212 (this study)
D. ancyla	185 (this study)			*D. pandora (MK)*	KU686374.1			*D. suzukii*	KF943819.1
D. atroscutellata	187 (this study)			*D. pandora (wRi)*	KU686368.1			*D. borealis*	FJ415471.1
D. prolaticilia	197 (this study)			*D. suzukii*	KF943818.1			*D. melanogaste r*	DQ842452.1
D. nr. *basimacula* #1	212 (this study)			*D. orientacea*	EU126285.1			*D. simulans*	DQ842435.1

D. nr. *basimacula* #2	213 (this study)			*D. munda*	EU126281.1			*D. simulans*	DQ842434.1
D. seclusa	221 (this study)			*D. testacea*	EU126277.1			*D. simulans*	DQ842433.1
D. nigrocirrus	244 (this study)			*D. innubila*	EU126276.1			*D. simulans*	DQ842432.1
D. recens	DQ842283.1			*D. neotestacea*	EU126271.1			*D. recens*	DQ842431.1
D. orientacea	DQ842282.1			*D. recens isolate 9*	DQ842394.1			*D. orientacea*	DQ842430.1

coxA		***fbpA***		***hcpA***		***ftsZ***		***gatB***	
***Wolbachia* Host**	**Accession Number**	***Wolbachia* Host**	**Accession Number**	***Wolbachia* Host**	**Accession Number**	***Wolbachia* Host**	**Accession Number**	***Wolbachia* Host**	**Accession Number**
D. neotestacea	DQ842281.1			*D. simulans isolate 26*	DQ842397.1			*D. neotestacea*	DQ842429.1
D. bifasciata	DQ842279.1			*D. melanogaster isolate 1*	DQ842415.1			*D. innubila*	DQ842428.1
D. innubila	DQ842280.1			*D. bifasciata isolate 5*	DQ842390.1			*D. bifasciata*	DQ842427.1
D. simulans	DQ842284.1			*D. pseudotakahashii (wPse)*	MF348260.1			*D. simulans*	KF717543.1
D. pseudotakahashii (wPse)	MF348256.1							*D. simulans*	KF717542.1
D. pandora (wRi)	KU686364.1							*D. simulans*	KF717541.1
D. pandora (MK)	KU686370.1							*D. orientacea*	EU126171.1
D. testacea	EU126220.1							*D. munda*	EU126167.1
								D. testacea	EU126163.1
								D. innubila	EU126162.1
								D. neotestacea	EU126157.1

								D. pandora (MK)	KU686373.1
								D. pandora (wRi)	KU686367.1
								D. simulans	KU255378.1
								D. simulans	KF717571.1
								D. simulans	KF717570.1

wspB		*wsp*							
**Wolbachia Host**	**Sample or Accession Number**	**Wolbachia Host**	**Sample or Accession Number**	**Wolbachia Host**	**Sample or Accession Number**	**Wolbachia Host**	**Sample or Accession Number**	**Wolbachia Host**	**Sample or Accession Number**
D. nr. *basimacula* #5	5 (this study)	*D. pseudoananassae (wPana)*	DQ412108.1	*D. orientacea*	DQ842466.1	*D. septentriosaltans (wSpt)*	AY620211.1	*D. ananassae*	AY858801.1
D. large spots	16 (this study)	*D. mauritiana (wNo)*	DQ412107.1	*D. innubila*	AY552553.1	*D. melanogaster*	KU870673.1	*D. pandora (wRi)*	KU686369.1
D. simulans	AJ580923.1	*D. simulans*	DQ842470.1	*D. innubila*	DQ842464.1	*D. melanogaster (wMel)*	DQ412100.1	*D. suzukii (72w)*	JX134954.1
D. simulans (wRi)	EU095939.1	*D. simulans*	DQ842469.1	*D. borealis*	FJ415468.1	*D. simulans (wMel)*	DQ412101.1	*D. suzukii (71w)*	JX134953.1
		D. kikkawai	JX134922.1	*D. munda*	EU126452.1	*D. melanogaster*	JF505383.1	*D. suzukii (70w)*	JX134952.1
		D. apicipuncta	JX134926.1	*D. pandora (MK)*	KU686375.1	*D. melanogaster*	LC108849.1	*D. paulistorum*	GQ924887.1
		D. innubila	AY552552.1	*D. recens*	AY154399.1	*D. melanogaster*	DQ842486.1	*D. simulans*	DQ842468.1
		D. Dorsigera	JX134935.1	*D. recens*	DQ842467.1	*D. melanogaster*	AY888036.1	*D. baimaii (wBai)*	DQ412091.1
		D. forficata	JX134928.1	*D. melanogaster*	AY888034.1	*D. paulistorum (wMel-like)*	KF289042.1	*D. rellima*	HM436813.1
		D. tetraspilota	JX134936.1	*D. septentriosaltans*	AY620214.1	*D. equinoxialis (wMel-like)*	KF289041.1		
		D. innubila	EU126447.1	*D. ambigua strain*	AY567540.1	*D. melanogaster*	AY888035.1		
		D. basimacula	JX134934.1	*D. tristis (wTri)*	AY567541.1	*D. neotestacea*	GU552301.1		
		D. basimacula	JX134933.1	*D. septentriosaltans (wSpt)*	AY620213.1	*D. takahashii (wTak)*	DQ412094.1		
		D. eurypeza strain	JX134927.1	*D. septentriosaltans (wSpt)*	AY620212.1	*D. pseudotakahashii (wPse)*	DQ412093.1		
		D. fundita	JX134924.1	*D. septentriosaltans (wSpt)*	AY620210.1	*D. bifasciata*	DQ842463.1		

		D. fundita	JX134929.1	*D. prosaltans (wPro)*	AY620208.1	*D. simulans (Riverside)*	EF423761.1		
wspB		***wsp***							
**Wolbachia Host**	**Sample or Accession Number**	**Wolbachia Host**	**Sample or Accession Number**	**Wolbachia Host**	**Sample or Accession Number**	**Wolbachia Host**	**Accession Number**	**Wolbachia Host**	**Sample or Accession Number**
		D. ancyla	JX134925.1	*D. arawakana (wWil)*	DQ412099.1	*D. simulans (Irvine 46)*	EF423760.1		
		D. dasycnemia	JX134915.1	*D. tropicalis (wWil)*	DQ412098.1	*D. simulans (Irvine 45)*	EF423759.1		
		D. dasycnemia	JX134920.1	*D. willistoni (wWil)*	DQ412097.1	*D. simulans (rvine 43)*	EF423758.1		
		D. dasycnemia	JX134912.1	*D. tropicalis (wAu)*	KF289039.1	*D. simulans (Irvine 42)*	EF423757.1		
		D. dasycnemia	JX134921.1	*D. willistoni (wWil)*	AY620229.1	*D. simulans (rvine 40)*	EF423756.1		
		D. redunca	JX134932.1	*D. willistoni (wWil)*	AY620228.1	*D. simulans (Irvine 36)*	EF423755.1		
		D. prodita	JX134931.1	*D. willistoni (wWil)*	AY620227.1	*D. simulans (Irvine 34)*	EF423754.1		
		D. dasycnemia	JX134919.1	*D. willistoni (wWil)*	AY620226.1	*D. simulans (Irvine 33)*	EF423753.1		
		D. dasycnemia	JX134918.1	*D. willistoni (wWil)*	AY620225.1	*D. simulans (Irvine 32)*	EF423752.1		
		D. dasycnemia	JX134917.1	*D. willistoni (wLag)*	AY620224.1	*D. simulans (Irvine 29)*	EF423751.1		
		D. dasycnemia	JX134916.1	*D. willistoni (wWil)*	AY620223.1	*D. simulans (Irvine 28)*	EF423750.1		
		D. dasycnemia	JX134914.1	*D. willistoni (wWil)*	AY620222.1	*D. simulans (Irvine 27)*	EF423749.1		
		D. dasycnemia	JX134913.1	*D. willistoni (wWil)*	AY620221.1	*D. simulans (Irvine 25)*	EF423748.1		
		D. dasycnemia	JX134911.1	*D. willistoni (wWil)*	AY620220.1	*D. simulans (Irvine 24)*	EF423747.1		
		D. dasycnemia	JX134910.1	*D. willistoni (wWil)*	AY620219.1	*D. simulans(Irvine 23)*	EF423746.1		

		D. nigrocirrus	JX134930.1	*D. willistoni (wWil)*	AY620218.1	*D. simulans (Irvine 21)*	EF423745.1		
		D. nikananu (wNik)	DQ412109.1	*D. paulistorum*	GQ924890.1	*D. simulans (Irvine 19)*	EF423744.1		
		D. sturtevanti (wStv)	AY620217.1	*D. paulistorum*	GQ924889.1	*D. simulans (Irvine 13)*	EF423743.1		
		D. sturtevanti (wStv)	AY620216.1	*D. yakuba*	AY291346.1	*D. simulans (Irvine 12)*	EF423742.1		
		D. sturtevanti (wStv)	DQ412110.1	*D. teissieri*	AY291347.1	*D. simulans (Irvine 11)*	EF423741.1		
		D. sturtevanti (wStv)	AY620215.1	*D. yakuba*	AY291348.1	*D. simulans (Irvine 10)*	EF423740.1		
		D. sechellia (wHa)	DQ412111.1	*D. paulistorum*	GQ924888.1	*D. simulans (Irvine 8)*	EF423739.1		
		D. fumipennis (wFum1)	AY620207.1	*D. septentriosaltans (wSpt)*	AY620209.1	*D. simulans (Irvine 5)*	EF423738.1		
		D. bicornuta (wBic)	DQ412092.1	*D. paulistorum (wAu-lik)*	KF289040.1	*D. simulans (Irvine 2)*	EF423737.1		
		D. neotestacea	DQ842465.1	*D. arawakana (wSpt)*	DQ412102.1	*D. simulans (Irvine 1)*	EF423736.1		
		D. neotestacea	EU126442.1	*D. ananassae (wSpt)*	DQ412103.1	*D. quadraria (wRi)*	DQ412096.1		
		D. orientacea	EU126456.1	*D. teissieri (wSpt)*	DQ412104.1	*D. ananassae (wRi)*	DQ412095.1		
		D. testacea	EU126448.1	*D. prosaltans*	DQ118779.1	*D. ananassae*	JQ518485.1		
