# Supplementary material for: The Transmission Patterns of the Endosymbiont Wolbachia within the Hawaiian Drosophilidae Adaptive Radiation
Source: Genes (Basel). 2023 Jul 27;14(8):1545. doi: 10.3390/genes14081545 (PMC10454618; doi:10.3390/genes14081545)
Supplement: Supplementary file 1 [file genes-14-01545-s001.zip › Table_S1_host_accessions.pdf]

Supplementary Table S1. National Center for Biotechnology Information (NCBI) accession numbers for Hawaiian Drosophilidae gene sequences selected for phylogenetic reconstruction of individual species having a verified *Wolbachia* infection, and genome accessions for outgroup taxa (also infected). Gene names and abbreviations are elongation factor 1-gamma (*EF1g*), glycerol-3-phosphate dehydrogenase (*Gpdh*), phosphoglucose isomerase (*Pgi*), and yolk protein 2 (*Yp2*).

**Sample ID**	**Species Name**	**Accession Number (NCBI)**					**Reference**
		EF1g	***Fz4***	***Gpdh***	***Pgi***	***Yp2***	
M09120	*Scaptomyza cyrtandrae*	JN815467.1	JN815636.1	JN815794.1	JN815958.1	JN816160.1	[1]
M09124	*Scaptomyza crassifemur*	JN815471.1	-----	JN815798.1	JN815962.1	JN816164.1	[1]
M09127	*Scaptomyza reducta*	JN815474.1	-----	JN815801.1	JN815965.1	JN816167.1	[1]
M09245	*Scaptomyza caliginosa*	JN815560.1	JN815714.1	JN815878.1	JN816041.1	JN816245.1	[1]
M09011	*Scaptomyza nasalis*	JN815359.1	JN815639.1	JN815722.1	JN815886.1	JN816052.1	[1]
M09015	*Scaptomyza undulata*	JN815363.1	JN815568.1	JN815724.1	JN815888.1	JN816056.1	[1]
M09066	*Drosophila perissopoda*	JN815413.1	JN815594.1	JN815751.1	JN815915.1	JN816106.1	[1]
M09028	*Drosophila quasiexpansa*	JN815376.1	JN815632.1	JN815790.1	JN815954.1	JN816153.1	[1]
M09075	*Drosophila atroscutellata*	JN815422.1	JN815603.1	JN815760.1	JN815924.1	JN816115.1	[1]
M09073	*Drosophila ancyla*	JN815420.1	JN815601.1	JN815758.1	JN815922.1	JN816113.1	[1]
M09228	*Drosophila nigrocirrus*	-----	JN815701.1	JN815864.1	-----	JN816230.1	[1]
M09230	*Drosophila* "large spots"	JN815548.1	JN815703.1	JN815866.1	JN816029.1	JN816232.1	[1]
M09095	*Drosophila kikiko*	JN815442.1	JN815621.1	JN815779.1	JN815943.1	JN816135.1	[1]
M09037	*Drosophila hawaiiensis*	JN815385.1	JN815573.1	JN815730.1	JN815894.1	JN816077.1	[1]
M09043	*Drosophila micromyia*	JN815390.1	JN815577.1	JN815734.1	JN815898.1	JN816083.1	[1]
M09096	*Drosophila engyochracea*	JN815443.1	JN815622.1	JN815780.1	JN815944.1	JN816136.1	[1]

Sample ID	Species Name	Accession Number (NCBI)					Citation
		EF1g	***Fz4***	***Gpdh***	***Pgi***	***Yp2***	
M09059	*Drosophila murphyi*	JN815406.1	JN815588.1	JN815745.1	JN815909.1	JN816080.1	[1]
M09061	*Drosophila ochracea*	JN815408.1	JN815590.1	JN815783.1	JN815911.1	JN816101.1	[1]
M09091	*Drosophila prolaticilia*	JN815438.1	JN815565.1	JN815719.1	JN815939.1	JN816131.1	[1]
M09063	*Drosophila prostopalpis*	JN815410.1	JN815592.1	JN815749.1	JN815913.1	JN816103.1	[1]
M09189	*Drosophila heteroneura*	JN815516.1	JN815675.1	JN815838.1	JN816002.1	JN816205.1	[1]
M09018	*Drosophila basimacula*	JN815366.1	-----	-----	-----	JN816059.1	[1]
M09030	*Drosophila seclusa*	JN815378.1	-----	-----	-----	JN816071.1	[1]
M09025	*Drosophila redunca*	JN815373.1	-----	-----	-----	JN816066.1	[1]
N/A	*Drosophila suzukii*	CAKG01035181.1	CAKG01024421.1	CAKG01004152.1	CAKG01035649.1	CAKG01056334.1	[2]
N/A	*Drosophila melanogaster*	NT_033777.3	NC_004354.4	NT_033779.5	NT_033778.4	NC_004354.4	[3]
N/A	*Drosophila simulans*	NT_479536.1	NC_029795.1	NT_479533.1	NT_479534.1	NC_029795.1	[4]
N/A	*Culex quinquefasciatus*	NW_001887359.1	-----	NW_001886703.1	NW_001886829.1	-----	[5]
N/A	*Aedes albopictus*	NW_017857380.1	-----	NW_017856043.1	NW_017857316.1	-----	[6]

References

1. Magnacca, K.N.; Price, D.K. Rapid adaptive radiation and host plant conservation in the Hawaiian picture wing *Drosophila* (Diptera: Drosophilidae). *Mol. Phylogenetics Evol.* **2015**, *92*, 226–242.
2. Ometto, L.; Cestaro, A.; Ramasamy, S.; Grassi, A.; Revadi, S.; Siozios, S.; Moretto, M.; Fontana, P.; Varotto, C.; Pisani, D.; et al. Linking genomics and ecology to investigate the complex evolution of an invasive *Drosophila* pest. *Genome Biol. Evol*. **2013**, *5*, 745–757.
3. dos Santos, G.; Schroeder, A.J.; Goodman, J.L.; Strelets, V.B.; Crosby, M.A.; Thurmond, J.; Emmeret, D.B.; Gelbart, W.M.; FlyBase Consortium. FlyBase: Introduction of the *Drosophila melanogaster* Release 6 reference genome assembly and large-scale migration of genome annotations. *Nucleic Acids Res*. **2015,** *43*, D690–D697.
4. Hu, T.T.; Eisen, M.B.; Thornton, K.R.; Andolfatto, P. A second-generation assembly of the *Drosophila simulans* genome provides new insights into patterns of lineage-specific divergence. *Genome Res*. **2013**, *23*, 89–98.
5. Arensburger, P.; Megy, K.; Waterhouse, R.M.; Abrudan, J.; Amedeo, P.; Antelo, B.; Bartholomay, L.; Bidwell, S.; Caler, E.; et al. Sequencing of *Culex quinquefasciatus* establishes a platform for mosquito comparative genomics. *Science* **2010,** 86–88.
6. Miller, J.R.; Koren, S.; Dilley, K.A.; Puri, V.; Brown, D.M.; Harkins, D.M.; Thibaud-Nissen, F.; Rosen, B.; Chen, X.G.; et al. Analysis of the Aedes albopictus C6/36 genome provides insight into cell line utility for viral propagation. *Gigascience* **2018,** *7*, 1–13.
