# Supplementary material for: The Transmission Patterns of the Endosymbiont Wolbachia within the Hawaiian Drosophilidae Adaptive Radiation
Source: Genes (Basel). 2023 Jul 27;14(8):1545. doi: 10.3390/genes14081545 (PMC10454618; doi:10.3390/genes14081545)
Supplement: Supplementary file 1 [file genes-14-01545-s001.zip › Table_S3_Stochastic_character_trait_mapping_model_fitting.pdf]

Supplementary Table S3. Record of Akaike information criterion (AIC) values obtained using the fitMK function in phytools v0.6-44 (Revell 2012) package in R to determine the best rates model to apply to each data set for stochastic character mapping analyses.

Models	Island of Collection		Ovipositional Substrate		Host Plant Family	
	Wolbachia in Hawaiian Drosophilidae	*Wolbachia* in Hawaiian Drosophilidae + mosquitoes	*Wolbachia* in Hawaiian Drosophilidae	*Wolbachia* in Hawaiian Drosophilidae + mosquitoes	*Wolbachia* in Hawaiian Drosophilidae	*Wolbachia* in Hawaiian Drosophilidae + mosquitoes
**Even Rates Model (ER)**	37.13527	41.55004	48.46443	53.6505	64.26912	72.05277
**Symmetric Rates Model (SYM)**	38.86977	43.77209	51.76297	57.1812	93.887	101.5524
**All Rates Different Model (ARD)**	44.94778	48.04271	59.44507	64.83332	136.63868	144.75148

References

Revell, L.J. Phytools: An R package for phylogenetic comparative biology (and other things). *Methods Ecol. Evol.* **2012**, *3*, 217–223.
