# Supplementary figures and images for: The Transmission Patterns of the Endosymbiont Wolbachia within the Hawaiian Drosophilidae Adaptive Radiation
Source: Genes (Basel). 2023 Jul 27;14(8):1545. doi: 10.3390/genes14081545 (PMC10454618; doi:10.3390/genes14081545)

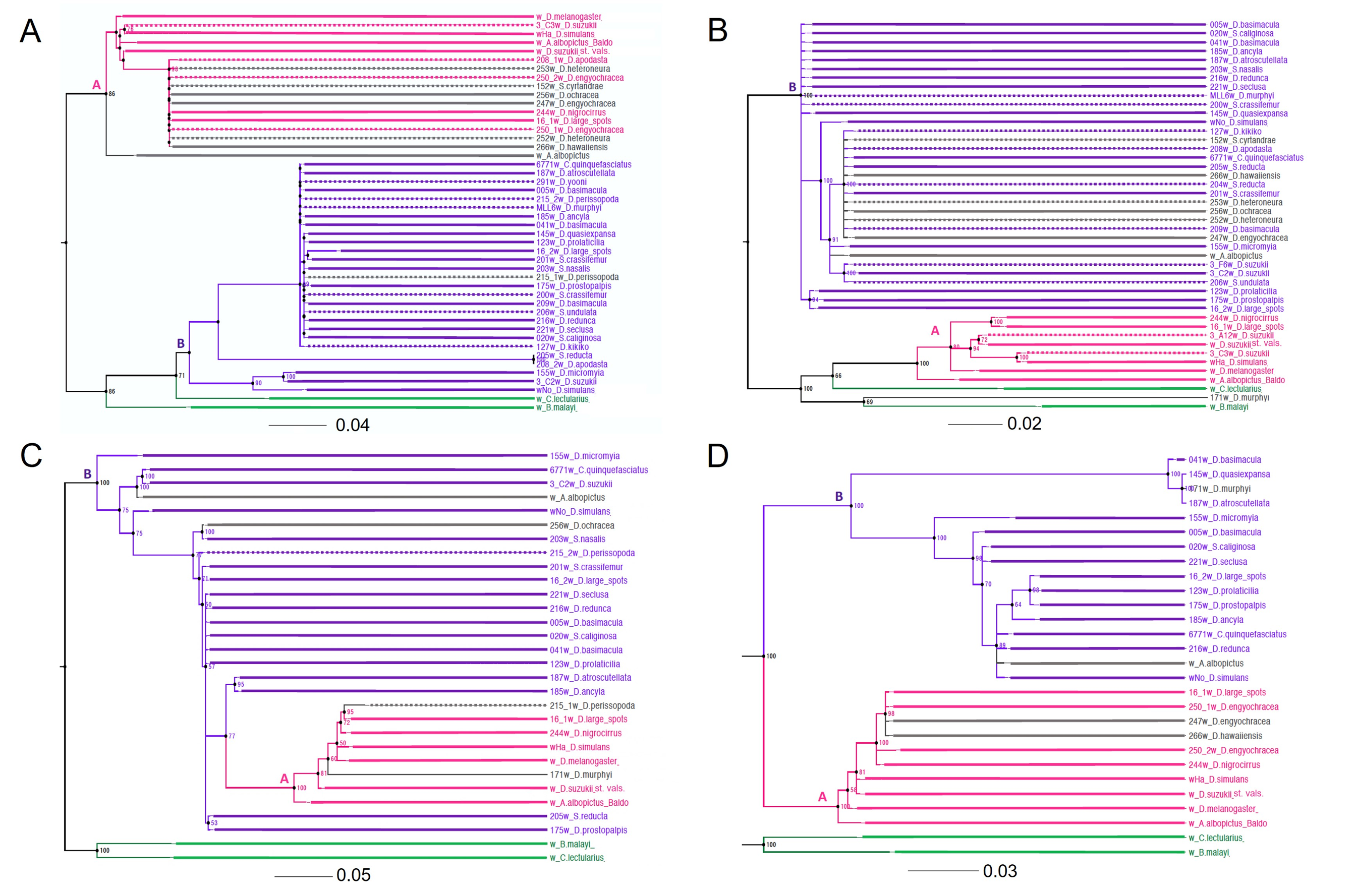

Supplement: Supplementary file 1 [file genes-14-01545-s001.zip › Figure S1.png]

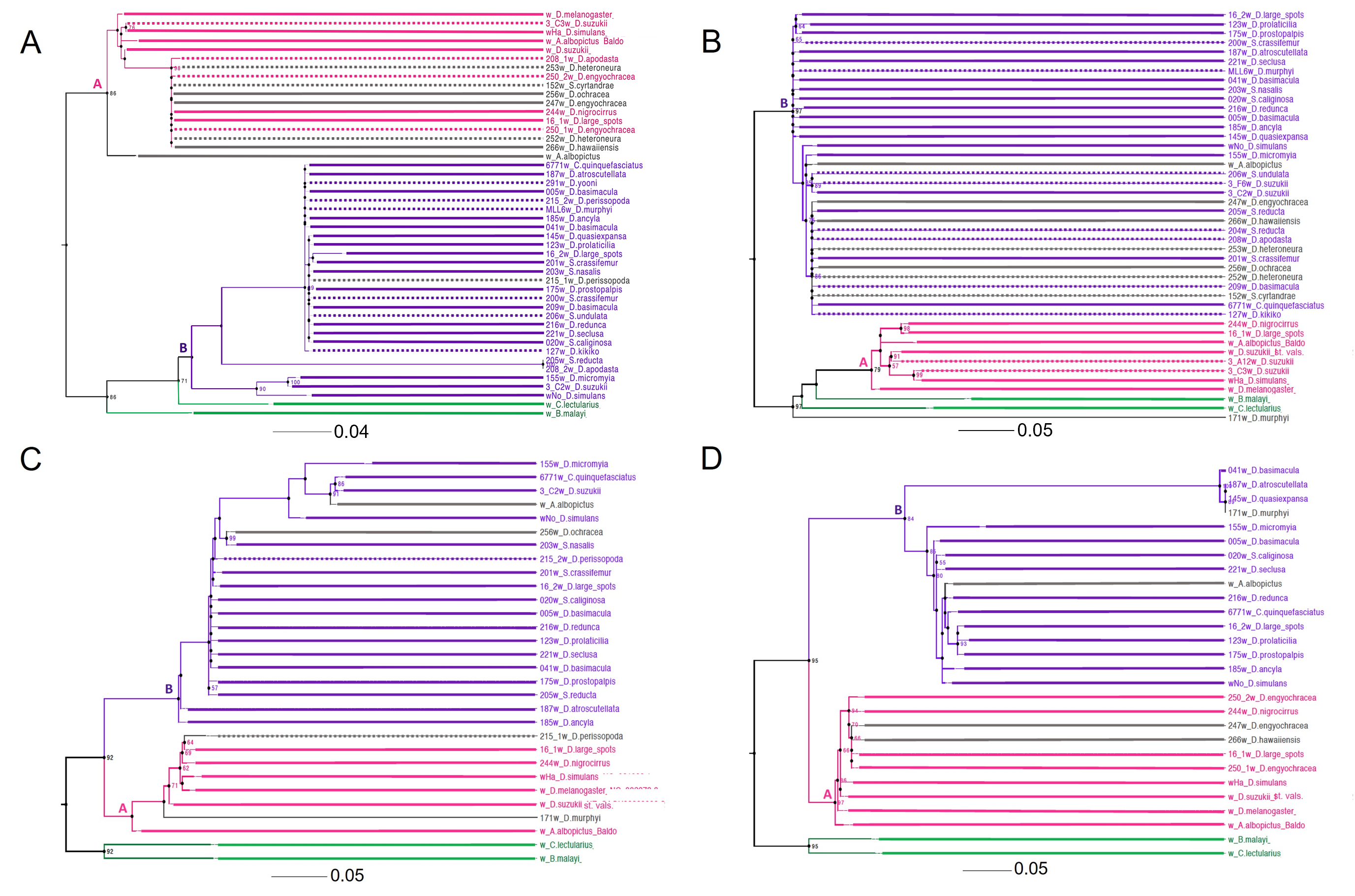

Supplement: Supplementary file 1 [file genes-14-01545-s001.zip › Figure S2.png]

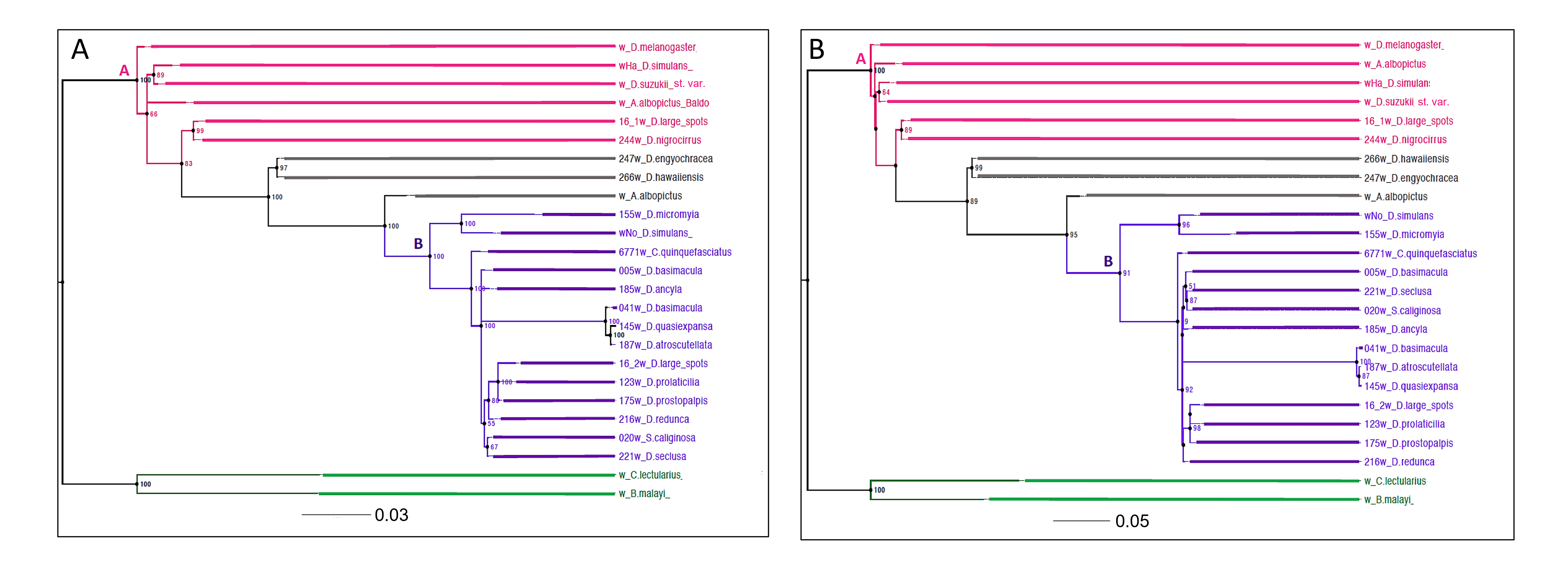

Supplement: Supplementary file 1 [file genes-14-01545-s001.zip › Figure S3.png]

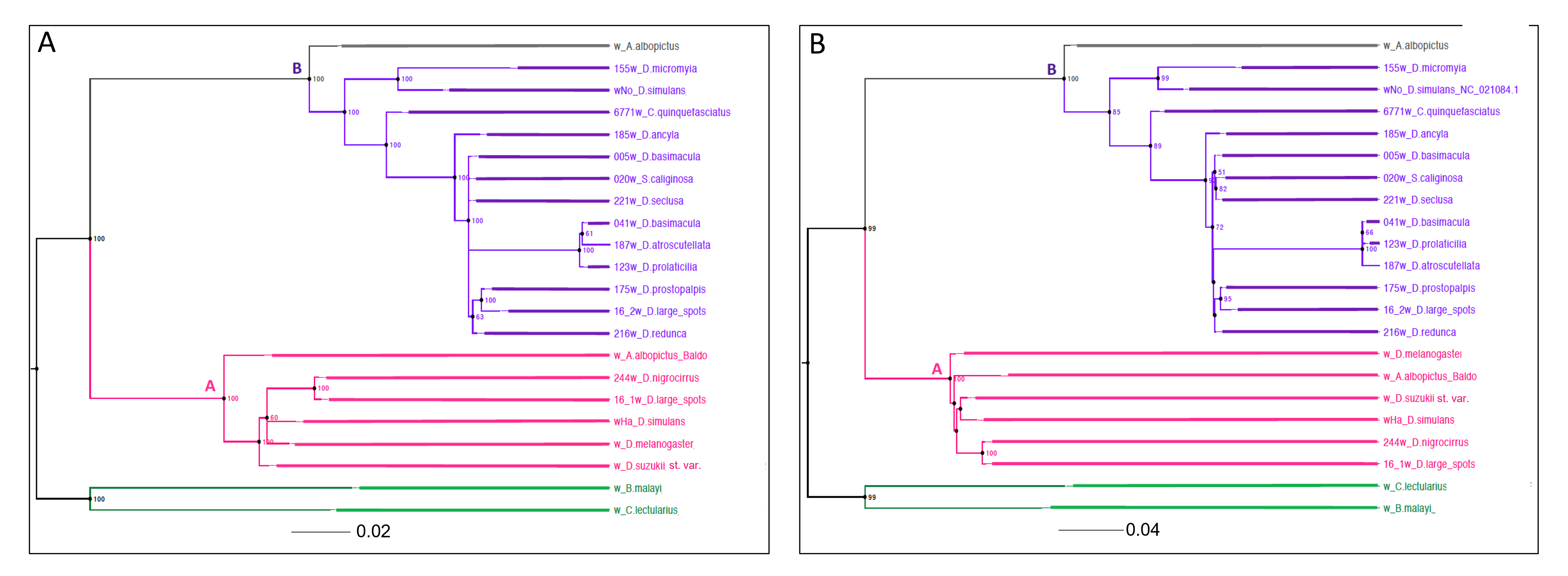

Supplement: Supplementary file 1 [file genes-14-01545-s001.zip › Figure S4.png]

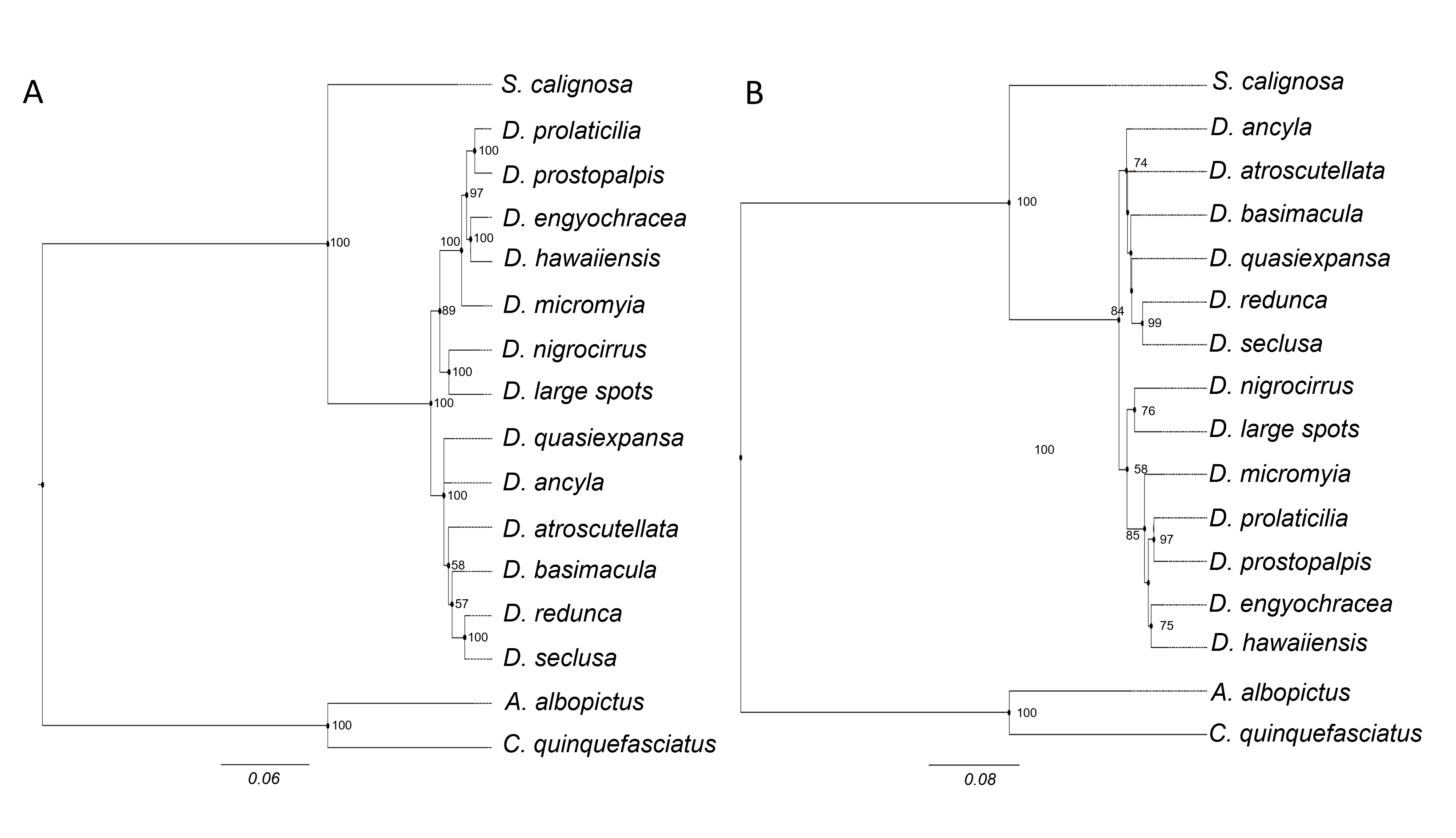

Supplement: Supplementary file 1 [file genes-14-01545-s001.zip › Figure S5.png]

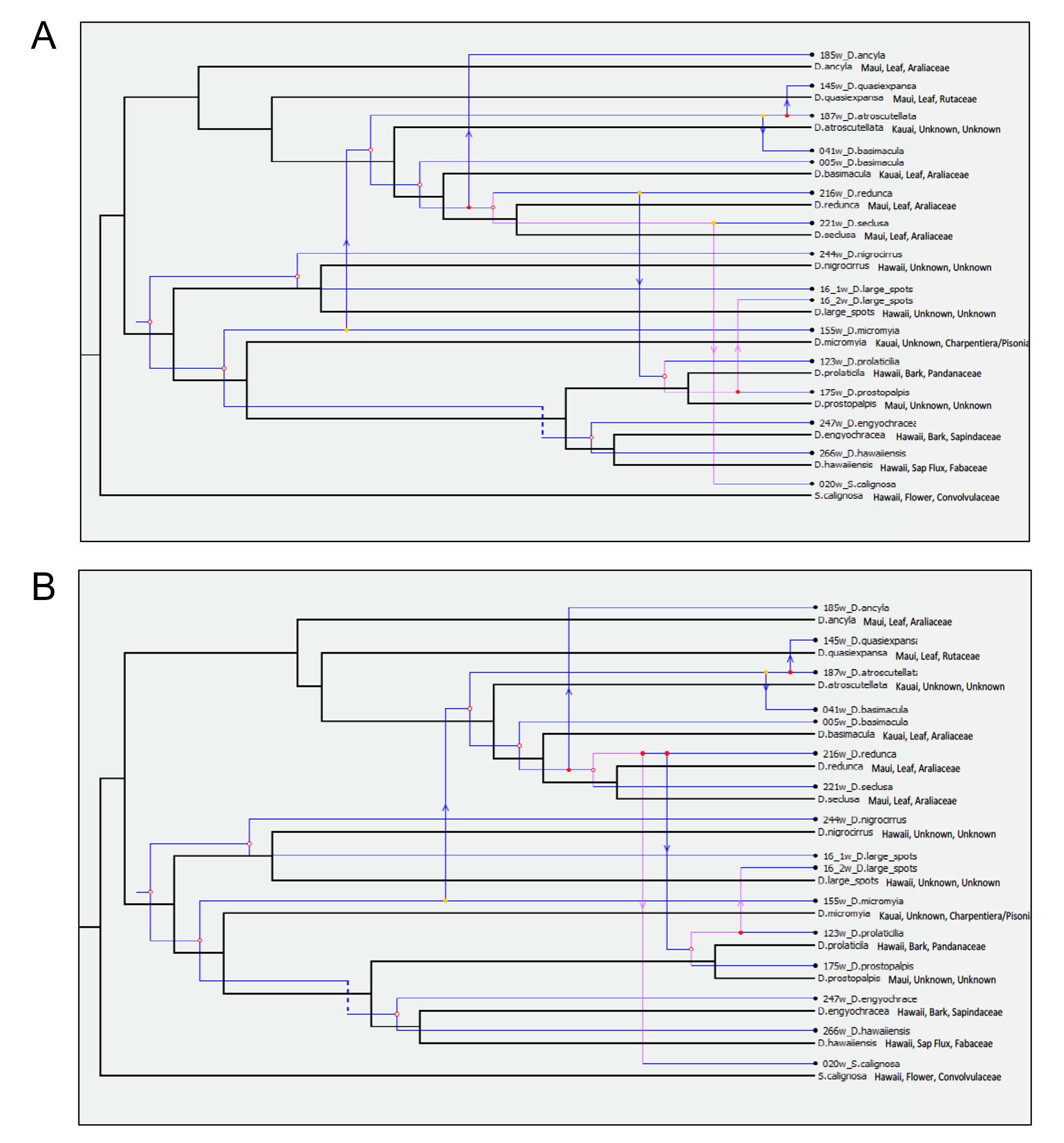

Supplement: Supplementary file 1 [file genes-14-01545-s001.zip › Figure S6.png]

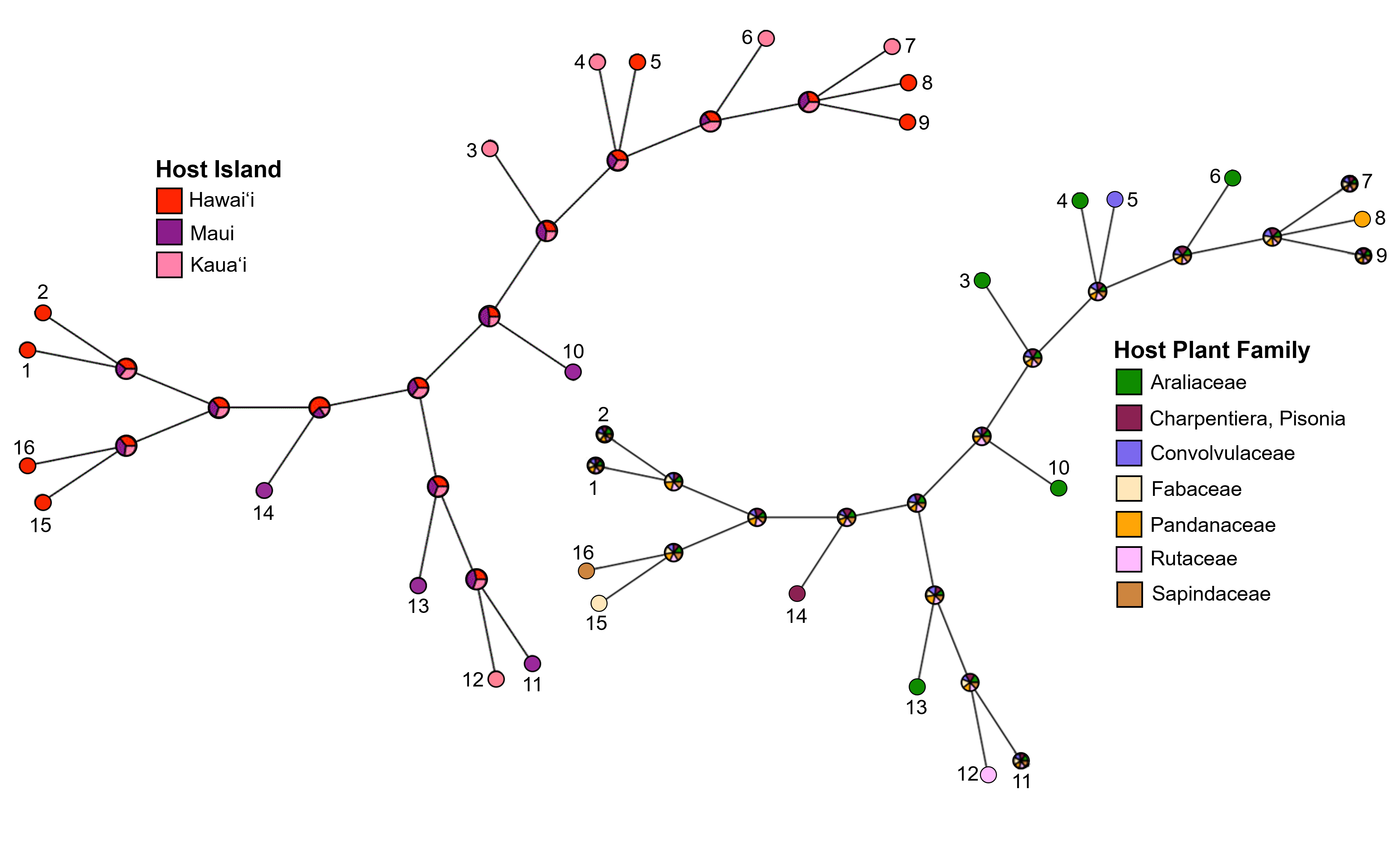

Supplement: Supplementary file 1 [file genes-14-01545-s001.zip › Figure S7.png]
